# Diagnosis of Endometriosis: Dual-Amplification Strategy Driven by Copper Nanoclusters

**DOI:** 10.1021/acs.analchem.6c00532

**Published:** 2026-05-14

**Authors:** Yu-Ling Wu, Hsu-Ching Yen, Pao-Ling Torng, Ja-An Annie Ho

**Affiliations:** † BioAnalytical Chemistry and Nanobiomedicine Laboratory, Department of Biochemical Science and Technology, 33561 National Taiwan University, Taipei 106319, Taiwan; ‡ Department of Obstetrics and Gynecology, National Taiwan University Hospital, Taipei 100217, Taiwan; § Department of Obstetrics and Gynecology, Tungs’ Taichung MetroHarbor Hospital, Taichung 435403, Taiwan; ∥ Department of Chemistry, National Taiwan University, Taipei 106319, Taiwan; ⊥ Center for Emerging Materials and Advanced Devices, National Taiwan University, Taipei 106319, Taiwan; # Center for Biotechnology, National Taiwan University, Taipei 106319, Taiwan; ∇ Professional Master’s Program of Biotechnology Management, School of Professional Education and Continuing Studies, National Taiwan University, Taipei 106319, Taiwan

## Abstract

Endometriosis is a prevalent gynecologic disorder associated with infertility and increased cancer risk, necessitating the development of sensitive and reliable diagnostic methods. Circulating microRNAs (miRNAs) have emerged as promising noninvasive biomarkers for early disease detection. However, low abundance and sequence similarity among miRNA family members hinder accurate detection. Herein, we first conducted differential expression analysis of publicly available miRNA-sequencing data sets to identify potential diagnostic biomarkers for endometriosis, from which miR-199a-5p was selected as a representative target. Building on this selection, we subsequently developed a highly sensitive and specific fluorescent biosensing platform for miR-199a-5p detection by integrating poly­(thymine) (polyT) DNA-templated copper nanoclusters (CuNCs) with a dual isothermal amplification strategy. The biosensing system utilizes a 3′-phosphorylated, biotinylated hairpin DNA probe immobilized on streptavidin-coated magnetic beads. Upon hybridization with the target miR-199a-5p, duplex-specific nuclease (DSN) mediates selective cleavage, enabling target recycling and simultaneously generating a 3′-hydroxyl terminus. This newly exposed terminus subsequently serves as a primer for terminal deoxynucleotidyl transferase (TdT)-catalyzed polyT elongation. The resulting polyT sequence functions as an effective scaffold for the *in situ* formation of copper nanoclusters (CuNCs), thus producing a label-free fluorescence signal within 2 h. In this design, magnetic beads not only facilitate efficient separation from serum matrices but also enhance reaction efficiency through surface-initiated enzymatic polymerization. As a result, the sensing platform exhibits excellent specificity, including reliable discrimination of single-base mismatches, and maintains robust performance in complex biological samples. This integrated platform, combining bioinformatic prescreening with a CuNCs-based sensing strategy, offers rapid, cost-effective, and label-free detection of miRNA, showing promise for early diagnosis and clinical monitoring of endometriosis.

## Graphical abstract

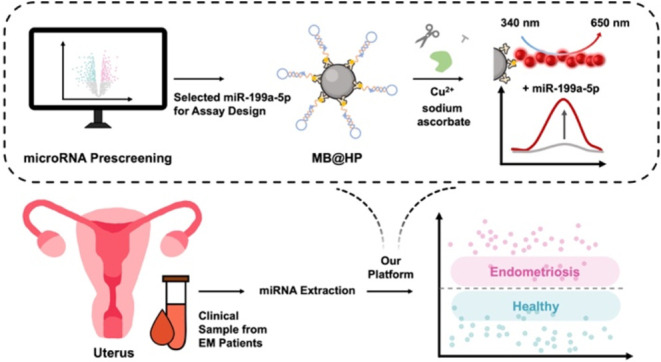

## Introduction

Endometriosis, characterized by the ectopic growth of endometrial tissue outside the uterus, is a chronic inflammatory disease associated with pelvic pain, conception difficulties, and, in severe cases, infertility.^1,2^ According to the World Health Organization, endometriosis affects approximately 10% of women of reproductive age worldwide, substantially impacting their quality of life.^3^ Laparoscopic examination followed by histological confirmation has long been considered the gold standard for the diagnosis of endometriosis.^4,5^ However, the invasive nature of laparoscopy, operative risks, limited availability of highly skilled surgeons, and significant financial burden have restricted its widespread application.^6^ In response, the European Society of Human Reproduction and Embryology (ESHRE) updated its guidelines in 2022, recommending clinical evaluation and imaging techniques as preliminary diagnostic approaches.^7^ Serum biomarkers, such as cancer antigen 125 (CA-125), have also been investigated as alternative diagnostic tools; however, their clinical utility remains limited due to poor specificity.^8,9^ Levels of CA-125 can be influenced by various gynecologic conditions and physiological changes during the menstrual cycle, reducing their reliability for diagnosing endometriosis.^10^ Consequently, accurate diagnosis continues to rely heavily on physician experience and the quality of imaging interpretation.

Currently, endometriosis is recognized as a chronic disease that requires lifelong management, primarily due to its high incidence of recurrence and frequent need for reoperation.^11^ This clinical reality highlights the pressing need for innovative and reliable biomarker-based diagnostic tools for initial diagnosis and long-term disease monitoring. Circulating microRNAs (miRNAs)—short, noncoding RNAs approximately 18–25 nucleotides—have emerged as promising candidates for disease biomarkers in recent years.^12^ Many studies have identified multiple miRNAs that are dysregulated in endometriosis and may be functionally linked to the invasiveness and proliferation of endometrial tissue.^13–15^ For example, Wang et al. employed microarray analysis and reported miR-122 and miR-199a-5p were upregulated, whereas miR-141–5p, miR-9, miR-524–3p, and miR-145–3p were downregulated in the serum of 60 patients.^14^ In a separate cohort, Lin et al. observed decreased levels of miR-17–5p and miR-424–5p in affected individuals.^16^ To systematically identify such disease-associated signatures, genome-wide profiling platforms, including microarrays and RNA sequencing, have been widely used to compare ectopic endometrium with eutopic or healthy endometrial tissue.^17,18^ Extending this approach, Tu et al. integrated miRNA sequencing data to evaluate immune microenvironment–related biomarkers in endometriosis, identifying differentially expressed miRNAs and statistically filtered biomarker candidates with diagnostic potential for endometriosis.^19^ Collectively, these studies support miRNAs as promising diagnostic biomarkers and highlight differential expression analysis as a rational strategy for target selection.

The distinctive characteristics of miRNAs, including their low abundance in serum and high sequence homology among family members, pose significant challenges for accurate detection and quantification in biosensing applications.^20^ At present, the clinical gold standard for nucleic acid detection is quantitative real-time polymerase chain reaction (qRT-PCR); however, its reliance on sophisticated instrumentation and precise thermal cycling constrains broader applicability.^21^ To address these limitations, a variety of biosensing strategies have been developed for the specific detection of disease-associated miRNAs. For example, Zhang et al. reported a machine-learning-assisted CRISPR/Cas13a-based fluorescent biosensor for identifying fecal extracellular vesicle miRNAs in noninvasive colorectal cancer detection.^22^ In a related effort, Yan et al. constructed a polymerase-based DNA molecular computing biosensor that encodes cancerous diagnostic valence (CDV) to perform weighted classification of non–small cell lung cancer from panels of CDV-informed miRNAs.^23^

Various isothermal nucleic acid amplification (INAA) strategies have been developed, utilizing a diverse array of enzymes, including nicking endonuclease,^24^ exonuclease,^25^ terminal deoxynucleotidyl transferase (TdT),^26^ DNA/RNA polymerase^27^ and duplex-specific nuclease (DSN).^28^ Among these, DSN has attracted particular attention for its remarkable ability to selectively cleave double-stranded DNA or DNA within the DNA/RNA heteroduplexes. This enables the efficient release of target miRNAs, which can be recycled to form an enzyme-cleavage cycle, thus generating a significant accumulation of products. When integrated with nanomaterials, DSN-based systems have facilitated the development of a wide range of fluorescent biosensors.^29–31^

Of the reported nanomaterials, DNA-templated fluorescent metal nanoclusters (NCs), particularly fluorescent gold nanoclusters (F-AuNCs), fluorescent silver nanoclusters (F-AgNCs), and fluorescent copper nanoclusters (F-CuNCs), have drawn increasing attention in recent decades.^32,33^ These NCs are composed of a few to several hundred metal atoms and exhibit notable properties, including strong emission, significant Stokes shift, high quantum yield, ease of functionalization, water solubility, and commendable biocompatibility. Compared to F-AuNCs and F-AgNCs, F-CuNCs offer additional advantages, such as simpler synthesis, tunable fluorescence emission,^34^ and great economic feasibility.^35,36^ The efficiency of CuNC formation has been found to correlate with both the length and the sequence of the DNA template,^37^ and a variety of scaffolds have been reported—including double-stranded DNA (especially AT/TA duplex),^38,39^ single-stranded poly­(thymine) (polyT) DNA^37,40^ and G-quadraplex structures.^41^ Given this background, terminal deoxynucleotidyl transferase (TdT), which catalyzes the addition of deoxyribonucleotides triphosphates (dNTP) to the 3′–OH terminus of DNA, offers a powerful means to generate controllable single-stranded products.^42^ By regulating the type and ratio of dNTPs, TdT-mediated amplification strategies have emerged as an effective approach to producing long polyT sequences, which serve as optimal templates for CuNCs synthesis.^43–46^

In this study, we first performed differential expression analysis of publicly available miRNA sequencing data sets from the Gene Expression Omnibus (GEO) database to identify biomarker candidates for endometriosis. Based on consistent dysregulation across data sets, we selected miR-199a-5p as a representative target for further investigation. Building on this data-driven selection, we developed a robust detection platform that integrates a hairpin-structured DNA probe immobilized on magnetic beads with a DSN-TdT dual isothermal amplification strategy, utilizing fluorescent copper nanoclusters (F-CuNCs) as the signal readout. Magnetic beads were incorporated not only to facilitate efficient separation from complex biological matrices, but also to enhance amplification performance by exploiting surface-initiated enzymatic polymerization (SIEP), which promotes TdT-catalyzed DNA chain elongation at solid interfaces.^47^ Altogether, this label-free and facile fluorescence-based assay establishes a proof-of-concept strategy that links bioinformatic biomarker prioritization with sensitive molecular detection, providing a promising framework for miRNA-based screening and risk stratification in endometriosis.

## Experimental Section

### Materials, Chemicals, and Apparatus

NaCl (sodium chloride), MgCl_2_ (magnesium chloride), KCl (potassium chloride), CO­(NH_2_)_2_ (Urea), C_6_H_7_NaO_6_ (Sodium l-Ascorbate), C_6_H_8_O_6_ (l-ascorbic acid), Na_2_HPO_4_ (sodium Phosphate dibasic), KH_2_PO_4_ (potassium phosphate monobasic), EDTA-Na_2_ (Ethylenediaminetetra Acetic Acid, disodium salt, dihydrate), CuSO_4_·5H_2_O (Copper­(II) sulfate pentahydrate), CHCl_3_ (chloroform), and DTT (dl-dithiothreitol) were purchased from Sigma-Aldrich (St. Louis, MO, USA). Tris-base (Tris (hydroxymethyl) methylaminomethane), Cu­(NO_3_)_2_ (Cupric nitrate,2.5-hydrate), and MOPS (3-[N-morpholino]-propanesulfonic acid) were acquired from J.T. Baker (Philipsburg, NJ, USA). Cu­(Cl)_2_·2H_2_O (Copper­(II) chloride, dihydrate) and H_3_BO_3_ (Boric acid) were purchased from Riedel-de-Haen (St. Louis, MO, USA). Terminal deoxynucleotidyl transferase (TdT), reaction buffer that contained 50 mM potassium acetate, 20 mM Tris-acetate, and 10 mM magnesium acetate at a pH of 7.9, and 0.25 mM CoCl_2_ (Cobalt­(II) chloride) solution were obtained from New England Biolabs. (Ipswich, MA, UK). Tetramethylethylenediamine (TEMED), polyacrylamide (29:1), and ammonium persulfate (APS) were acquired from MDBio Inc. (Taipei, Taiwan). Streptavidin-modified magnetic beads (100 nm) were obtained from Ocean NanoTech (San Diego, CA, USA). Duplex-specific nuclease (DSN) was acquired from Evrogen Joint Stock Company (Moscow, Russia). SYBR Gold, GeneRuler ultralow range DNA ladder, DNA Gel Loading dye (6x), dTTP, and commercial qRT-PCR kits (TaqMan MicroRNA Reverse Transcription Kit, MicroRNA Assay, and Universal PCR Master Mix) for miR-199a-5p were obtained from Thermo Fisher (Waltham, MA, USA). The Human miRNeasy serum/plasma kit for miRNA purification was obtained from Qiagen (Qiagen, Hilden, Germany). Dr. Pao-Ling Torng provided the clinical serum samples from National Taiwan University Hospital, Taipei, Taiwan (IRB no. 110–006-E). All aqueous solutions were prepared using distilled and deionized water (ddH_2_O; resistivity ≈ 18.2 MΩ·cm at 25 °C) purified with a Milli-Q water purification system (Burlington, MA, USA). All the oligonucleotides were synthesized and purified by Integrated DNA Technologies (Coralville, IA, USA). The sequences are listed in Table S1.

Polyacrylamide gel electrophoresis (PAGE) was operated on Mini-PROTEAN Tetra Cell (Bio-Rad, Hercules, CA, USA). The polyacrylamide gel was imaged by a ChemiDoc Touch Imaging System (Bio-Rad, Hercules, CA, USA). The temperature of the amplification reaction was controlled by Touch Thermal Cycler (Bio-Rad, Hercules, CA, USA). The UV–vis absorption spectra and fluorescence measurements of CuNCs were recorded with Varioskan LUX Multimode Microplate Reader (Thermo Fisher Scientific, Rockford, IL, USA). Centrifugation of the serum sample was carried out with a Z326 K Universal Refrigerated Centrifuge (Hermle, Baden-Württemberg, Germany). The size distribution and zeta potential of the conjugate formed by a hairpin probe and magnetic beads were investigated using a Zetasizer Nano ZS (Malvern, Worcestershire, UK). The morphology of magnetic beads and magnetic beads-copper nanoclusters images were characterized by H-7650 Transmission Electron Microscopy (Hitachi, Japan).

### MiRNA Differential Expression Analysis

The miRNA expression profiles of endometriosis patients were obtained from the Gene Expression Omnibus (GEO) under accession numbers GSE153813 (*N* = 8; 5 patients and 3 controls) and GSE105765 (*N* = 16; 8 patients and 8 controls). In both data sets, individuals without uterine or pelvic pathology were used as the control group. Differential expression analysis between endometriosis and control samples was performed using the DESeq2 package (v.1.42.1) in R software (v.4.3.2). Adjusted *P* < 0.05 and |log2­(fold change)| ≥ 0.5 were used as the thresholds to identify differentially expressed miRNAs between endometriosis patient and control samples.

### Hairpin Probe Design and Optimization

Hairpin probe candidates were first designed and evaluated using NUPACK. The predicted secondary structure and stability were tuned by varying the stem and loop lengths systematically. The corresponding oligonucleotides were then experimentally tested. For structure formation, selected hairpin DNA sequences were heated and slowly cooled to room temperature to promote intramolecular folding into the predicted hairpin structures. Each hairpin probe was mixed with the complementary target miR-199a-5p in 1× DSN buffer (50 mM Tris-HCl, 1 mM DTT, 5 mM MgCl_2_, pH 8.0) at a final concentration of 200 nM for both strands, followed by the addition of 0.5 U DSN to give a total reaction volume of 10 μL.

To validate the DSN-assisted digestion, a 15% denaturing polyacrylamide gel electrophoresis (PAGE) assay containing 7 M urea was performed in 0.5× TBE buffer (50 mM Tris-base, 50 mM boric acid, and 1 mM EDTA). The gel was prerun at 35 V for 30 min at 50 °C, followed by electrophoresis at 70 V for 80 min at 50 °C after loading the DSN-cleaved products. The denaturing gel was stained with SYBR Gold for 15 min and visualized using a ChemiDoc Imaging System.

### Specificity of Hairpin Probe

To evaluate sequence selectivity toward miR-199a-5p, the hairpin probe and a corresponding linear probe were separately incubated with target DNA analogs bearing one, two, or three mismatched bases relative to the entirely complementary DNA-199a-5p sequence. Each probe–target pair was prepared in 1× DSN buffer at a final concentration of 200 nM, followed by the addition of 0.5 U DSN to a total reaction volume of 10 μL. Reactions were carried out at 37 °C for 1 h, and the products were subsequently analyzed by denaturing PAGE to assess DSN cleavage efficiency. We also tested the selectivity of hairpin DNA against nontarget miRNAs, including miR-21, let-7a, miR-141, and miRNA-200a following the same procedure.

### Melting Curve Analysis of the Hairpin Probe and the Hairpin Probe/Target Duplex

To determine the thermal stability of the hairpin probe and its duplex with the target, either the hairpin probe alone or the hairpin probe prehybridized with the target was prepared in 1× DSN buffer at a final strand concentration of 200 nM. After annealing, SYBR Green was added to each sample at a final dilution of 1:10,000 (v/v) from the stock solution. The dye-DNA mixtures (total volume 20 μL) were incubated at room temperature for 15 min to allow dye binding. Melting experiments were then performed on a real-time PCR thermocycler by continuously heating the samples from room temperature to 95 °C while monitoring fluorescence. Melting curves were generated from the fluorescence–temperature profiles, and the *T*_m_ values for the hairpin alone and the hairpin–target duplex were extracted from the derivative plots. A DNA analogue of miR-199a-5p (DNA-199a-5p) was used in place of the RNA target in this experiment to enable efficient staining with SYBR Green, a DNA-duplex–intercalating dye.

### Preparation and Characterization of the Conjugated hDNA on MB (MB-hDNA)

Before the conjugation of the biotinylated hairpin DNA probe to the streptavidin-functionalized magnetic beads (SA-MBs), 10 μL of the SA-MB (∼1.38 × 10^8^ particles/μL) were first magnetically separated to remove the storage buffer, washed with PBS buffer (137 mM NaCl, 2.7 mM KCl, and 10 mM phosphate buffer at a pH of 7.4), and resuspended in 10 μL PBS. Subsequently, 40 μL of 250 nM hairpin probe was added and then incubated for 30 min at room temperature. The excess unconjugated probes were removed after magnetic separation. Finally, the prepared MB-hDNA were washed three times with PBS and resuspended in PBS before use. The size distribution and zeta potential of MB-hDNA were investigated using a Malvern Zetasizer Nano ZS. The unconjugated probes were stained with SYPR GOLD, and fluorescence intensities were obtained using a microplate reader. A calibration curve between fluorescence and various concentrations of the probe was established to quantify the unconjugated probes, thereby determining the number of conjugated probes on each magnetic bead using the following eq 1

1 $$\mathrm{DNA}\;\mathrm{strands}/\mathrm{MB}=\frac{C_{\mathrm{probe}}\times 10^{-9}\times V_{\mathrm{probe}}\times 10^{-6}\times N_{A}}{V_{\mathrm{MB}}\times 1.38\times 10^{8}}$$

where *C*_probe_ is the concentration of conjugated probe (nM), *V*_probe_ is the volume of probe solution added (μL), *V*_MB_ is the volume of MB solution (μL), and *N*_A_ is the Avogadro constant.

### Synthesis and Characterization of Copper Nanoclusters (CuNCs)

For the synthesis of DNA-templated CuNCs, solutions containing 4 μM T60 in 10 mM MOPS buffer (150 mM NaCl, pH 7.9) were prepared. After the addition of Cu^2+^ and freshly prepared ascorbate salt, the reaction mixtures (final volume of 50 μL) were incubated in the dark at room temperature for 5 min. The resulting CuNCs were characterized using UV–vis spectroscopy and fluorescence spectroscopy with a Varioskan LUX Multimode Microplate Reader. The fluorescence emission of the samples was recorded at an excitation wavelength of 340 nm and an emission wavelength of 650 nm. To determine the optimal conditions for the formation of DNA-templated CuNCs, three different Cu^2+^ sources, CuSO_4_, Cu­(NO_3_)_2,_ and CuCl_2_, were evaluated along with two reducing agents: sodium ascorbate and ascorbic acid. In addition, various concentrations of CuSO_4_ (100, 200, and 300 μM) and sodium ascorbate (1, 2, 3 mM) were tested to optimize the reaction parameters.

The effect of cations in the TdT reaction buffer was evaluated using DNA-templated CuNCs prepared under different conditions: MOPS buffer alone, MOPS buffer supplemented with 10 mM Mg^2+^, 50 mM K^+^, and 0.5 mM Co^2+^, TdT reaction buffer, and a 1:1 (v/v) mixture of MOPS and TdT buffers. The temporal stability of DNA-templated CuNCs under optimized buffer conditions was assessed by recording fluorescence after 2.5, 5, 10, 15, and 30 min of incubation.

### Validation of DSN Reaction on Magnetic Beads

The hybridization between target miR-199a-5p and the hairpin probe conjugated to magnetic beads was previously validated using 15% native PAGE in 0.5× TBE buffer (50 mM Tris-base, 50 mM Boric acid, 1 mM EDTA). Electrophoresis was carried out at 70 V for 120 min. Following separation, the gel was stained with SYBR Gold for 15 min, and the image was captured using a ChemiDoc Imaging System.

### Optimization of the Reaction Conditions of TdT-Mediated Polymerization

The synthesized biotinylated linear DNA probe (Pcut), which contains a free 3′-hydroxyl group, was immobilized onto magnetic beads to generate the MB–Pcut complex as described above. To optimize the amount of immobilized probe, different volumes of the MB–Pcut suspension (10, 15, or 20 μL) were added to the reaction mixture, and the corresponding probe densities on the beads (2024, 3036, or 4048 DNA strands per MB) were evaluated under these conditions.

The TdT reaction was then optimized by varying the incubation times (0.5, 1.5, and 2.5 h) using 20 U TdT and 3 mM dTTP. The concentration of dTTP was further optimized at 2, 3, 4, and 5 mM (20 U TdT, 1.5 h), while the amount of TdT was varied from 10 to 25 U (4 mM dTTP, 1.5 h). Subsequently, 6 μL of 10 mM MOPS buffer, 1.2 μL of 10 mM CuSO_4_, and 1.2 μL of 100 mM sodium ascorbate were added to the TdT product, yielding a final reaction volume of 60 μL. The fluorescence emission spectra were recorded with excitation at 340 nm and emission scanned from 520 to 800 nm, with the maximum intensity observed at 650 nm.

### Procedure for the Detection of miR-199a-5p

The as-prepared MB-hDNA was resuspended in DSN reaction buffer (50 mM Tris–HCl, 1 mM DTT, 5 mM MgCl_2_, pH 8.0) and incubated with various concentrations (0, 0.1, 1, 10, 50, 100, and 200 nM) of target miR-199a-5p and 0.5 U DSN in a final volume of 30 μL at 37 °C for 1 h. After magnetic separation, the beads were resuspended in TdT reaction buffer (50 mM potassium acetate, 20 mM Tris-acetate, 10 mM magnesium acetate and CoCl_2_, pH 7.9), and further incubated for 1.5 h with an addition of 20 U TdT and 4 mM dTTP (final volume: 50 μL). Finally, 6 μL of 10 mM MOPS buffer, 1.2 μL of 10 mM CuSO_4_, and 1.2 μL of 100 mM sodium ascorbate were added to reach a total reaction volume of 60 μL. The fluorescence emission spectra were recorded with excitation at 340 nm and emission scanned from 520 to 800 nm. We also tested the selectivity of the proposed sensing platform against nontarget miRNAs, including miR-21, let-7a, miR-141 and miRNA-200a, at 100 nM each, following the same procedure.

### Detection of miR-199a-5p in Serum Samples from Endometriosis Patients and Controls

The human serum samples were collected under the guidance of Dr. Pao-Ling Torng at the National Taiwan University Hospital. RNA extraction from these samples was performed using the miRNeasy serum/plasma kit (QIAGEN), following the manufacturer’s supplementary protocol. Briefly, 200 μL of serum was thoroughly mixed with 1 mL of QIAzol lysis reagent and incubated for 5 min to disrupt nucleoprotein complexes. Subsequently, 0.2 mL of CHCl_3_ was added, vortexed for 15 s, and incubated at room temperature for 3 min. The microRNA-containing aqueous phase was obtained by centrifugation at 12,000*g* for 15 min at 4 °C. The upper aqueous layer was transferred to a new collection tube, and 1.5 volumes of 95% ethanol were added. A 700 μL aliquot of the mixture was then applied to a spin column provided in the kit and centrifuged at 10,000*g* for 15 s, followed by two additional washes using the supplied buffers. Finally, the purified microRNA was eluted with 14 μL of double-distilled water and stored at −20 °C until further analysis.

### MiR-199a-5p Quantification Using TaqMan MicroRNA Assays

Quantitative real-time PCR (qRT-PCR) was performed using TaqMan MicroRNA Assays according to the manufacturer’s protocol. Briefly, reverse transcription (RT) reactions were prepared in a total volume of 15 μL, containing 5 μL of RNA sample, 3 μL of RT primer, and 7 μL of RT master mix (0.15 μL of 100 mM dNTPs, 1 μL of 50 U/μL reverse transcriptase, 1.5 μL of 10× RT buffer, 0.19 μL of 20 U/μL RNase inhibitor, and 4.16 μL of double-distilled water). The RT reactions were carried out at 16 °C for 30 min, 42 °C for 50 min, and 85 °C for 5 s, followed by a hold at 4 °C. Subsequently, PCR amplification was performed in a 20 μL reaction mixture containing 1.33 μL of RT product, 1 μL of 20× TaqMan MicroRNA Assay, 10 μL of TaqMan Universal PCR Master Mix, and 7.67 μL of double-distilled water. The thermal cycling conditions were as follows: an initial denaturation at 95 °C for 10 min, followed by 40 cycles of denaturation at 95 °C for 15 s and annealing/extension at 60 °C for 60 s. Each reaction was conducted in duplicate, and the threshold cycle (Ct) values were determined automatically by the instrument software.

## Results and Discussion

### Identification of Dysregulated MiRNAs in Endometriosis

Prior to detection assay, bioinformatic analysis of publicly available data sets was first performed to identify miRNAs that are differentially expressed in endometriosis, from which miRNA candidates with potential diagnostic relevance from each miRNA data set were compared. Briefly, bioinformatic analysis of publicly available data sets was first performed to identify miRNAs and their target mRNAs that are differentially expressed in endometriosis, from which candidate biomarkers with potential diagnostic relevance were prioritized (Figure 1A). To understand and identify circulating miRNAs that are dysregulated in endometriosis, we analyzed two independent plasma-based miRNA-sequencing data sets from GEO. In GSE105765, differential expression analysis between endometriosis patients (*n* = 8) and controls (*n* = 8) identified 336 significantly dysregulated miRNAs, including 171 upregulated and 165 downregulated species (Figures 1A and S1). In GSE153813, an analogous comparison between endometriosis patients (*n* = 5) and controls (*n* = 3) revealed 30 dysregulated miRNAs, comprising 21 upregulated and 9 downregulated miRNAs (Figures 1B and S2). To focus on miRNAs with consistent expression changes, we overlapped the significantly upregulated and downregulated miRNAs from both miRNA-sequencing data sets. This comparison yielded nine commonly upregulated miRNAs (miR-145–3p, miR-136–3p, miR-143–3p, miR-199a-5p, miR-493–5p, miR-889–3p, miR-509–3–5p, miR-23a-3p, and miR-381–3p) and two commonly downregulated miRNAs (miR-375 and miR-92a-3p), which were concordantly dysregulated across both cohorts (Figure 1C).

**Figure 1:**
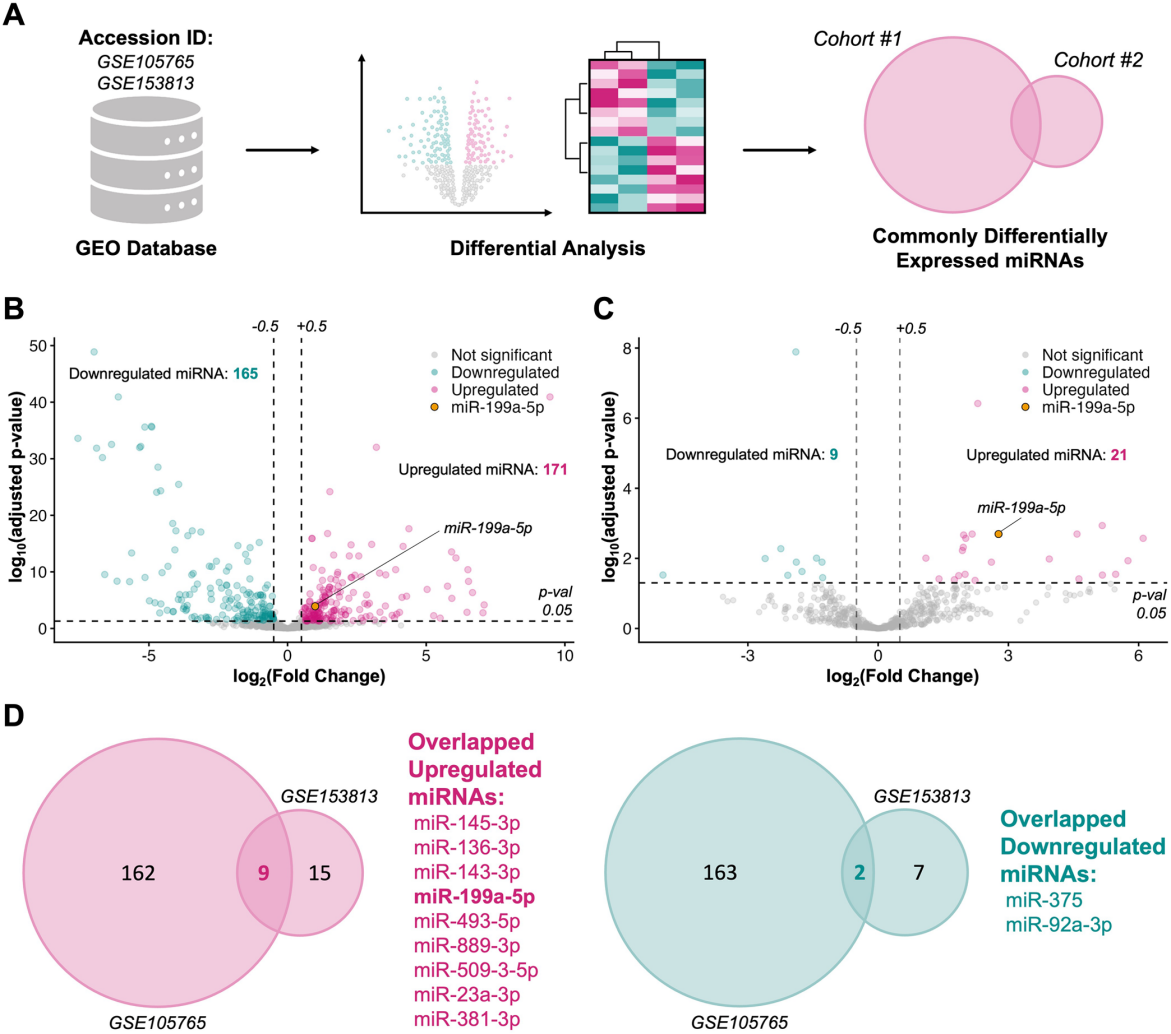
Dysregulated miRNA expression profiles in endometriosis. (A) Differential expression analysis of miRNA-sequencing profiles from GEO data sets (GSE105765 and GSE15813) comparing healthy controls and patients with endometriosis. (B) Differentially expressed miRNAs identified in the GSE105765 data set. (C) Differentially expressed miRNAs identified in the GSE153813 data set. (D) Venn diagram showing the overlap of commonly upregulated and downregulated miRNAs between the two endometriosis data sets.

Among these consistently upregulated candidates, miR-199a-5p was selected as the focus of our initial investigation based on evidence from prior serum studies. Elevated levels of miR-199a-5p have been repeatedly reported in women with endometriosis, supporting its potential diagnostic relevance.^14,48–51^ In a microarray-based study by Wang et al., miR-199a-5p was identified as upregulated in patients’ serum. Moreover, a combined miRNA panel consisting of miR-122, miR-199a-5p, miR-145, and miR-542 achieved an area under the curve (AUC) of 0.994 for endometriosis diagnosis.^14^ Consistent findings were reported by Maged et al., who observed upregulation of miR-122 and miR-199a-5p, with miR-122 showing 95.6% sensitivity and 91.4% specificity, and miR-199a-5p achieving 100% sensitivity and 100% specificity.^50^ Building on this converging evidence and our differential expression analysis, we selected miR-199a-5p as the primary target for developing and validating the detection platform. Our goal is to establish a more efficient method for determining serum miR-199a-5p levels and to evaluate its relevance to endometriosis in a proof-of-concept study. With future large-scale validation, this approach may enable the development of standardized analytical tools for early detection, preoperative decision-making, and longitudinal disease monitoring.

### Assay Principle

The principle of our detection platform is illustrated in Figure 2. Based on the miRNA prescreening (Figure 1), miR-199a-5p was selected as a representative biomarker to establish the proof-of-concept detection strategy. The complete detection workflow, together with the corresponding time requirements for each step, is summarized in Table S2.

**Figure 2:**
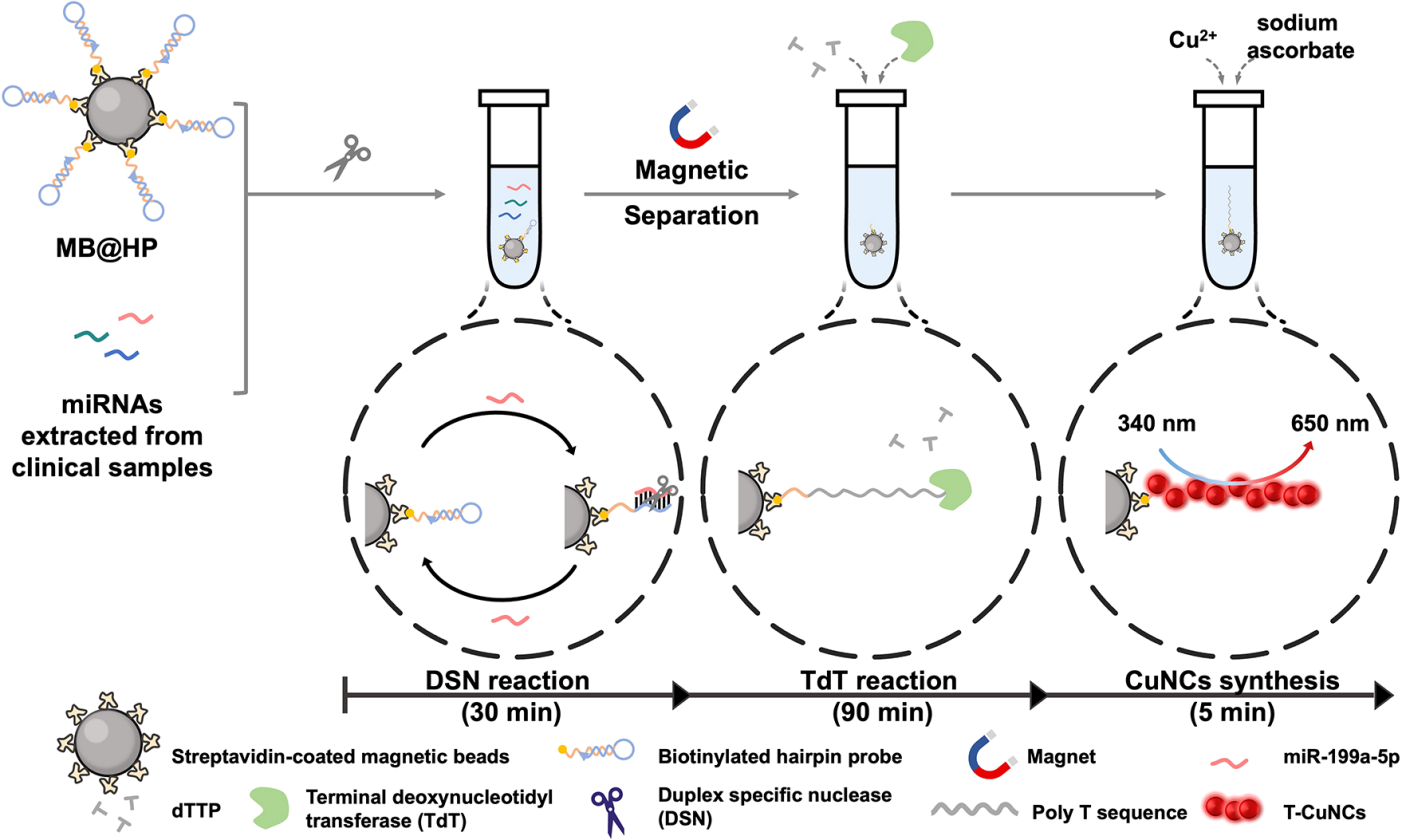
Schematic representation of the detection mechanism for endometriosis-associated miRNA. The schematic illustrates the overall detection workflow, in which the capture probe specifically hybridizes with target miR-199a-5p, initiating TdT-mediated extension and signal amplification. The resulting elongated products generate fluorescence signals proportional to miR-199a-5p concentration, enabling sensitive, quantitative detection.

In this system, a biotinylated hairpin-structured probe DNA (hDNA) is immobilized onto streptavidin-functionalized magnetic beads (SA-MB) via streptavidin–biotin interaction. This hDNA adopts a “balloon-like” conformation that preserves its structural integrity against duplex-specific nuclease (DSN) digestion and is further designed with a phosphorylated 3′-terminus to prevent terminal deoxynucleotidyl transferase (TdT)-mediated extension. Upon the introduction of target miR-199a-5p, the hairpin structure is opened and hybridized with miR-199a-5p, thus initiating the DSN-assisted target recycling process. The resulting heteroduplex (hDNA/miR-199a-5p) is recognized explicitly by DSN, which selectively digests the DNA strand and releases the intact miRNA, triggering the next reaction cycle. Following magnetic separation, the remaining hDNA with an exposed 3′-hydroxyl terminus is effectively elongated into a long polyT sequence in the presence of TdT and dTTP. After another magnetic separation, the addition of copper sulfate and sodium ascorbate facilitates the in situ formation of fluorescent copper nanoclusters (CuNCs). The fluorescence intensity of CuNCs exhibits a positive correlation with the concentration of target miR-199a-5p, allowing quantitative determination of miR-199a-5p levels in endometriosis serum samples.

### The “Balloon-like” Probe hDNA Design

In designing our biosensing platform, we did not aim to simply refine an existing DSN-based assay, but rather to systematically establish design principles for hairpin-structured probes used in DSN-mediated amplification reactions. Particular emphasis was placed on the structural configuration of the hairpin probe, as probe architecture plays a decisive role in balancing enzymatic accessibility, background suppression, and signal amplification efficiency. Accordingly, we focused on the rational design of a “balloon-like” hairpin structure that preserves probe integrity in the absence of the target while enabling controlled activation upon target hybridization. This design strategy provides a generalizable framework for probe engineering in DSN-assisted biosensing systems, extending beyond empirical optimization and offering broader applicability to related nucleic-acid–based amplification platforms.

Our probe comprises a linker region conjugated to the magnetic bead, a loop sequence complementary to the target miR-199a-5p, and a stem region forming an intramolecular duplex. Considering the specific cleavage characteristics of DSN, two critical design principles were emphasized: (1) the hairpin structure must efficiently unfold upon hybridization with target miR-199a-5p to form a duplex recognizable by DSN for subsequent digestion; and (2) in the absence of the target, the hairpin probe should maintain structural stability and resist DSN cleavage to minimize undesired background signals.

To ensure the reliability of the probe design and achieve the anticipated performance, our initial step was to determine the optimal number of base pairs in the stem region. Previous studies have shown that DSN preferentially cleaves DNA duplexes longer than eight base pairs.^52,53^ Based on this finding, four hairpin-structured DNAs with varying stem lengths—6, 7, 8, and 9 base pairs—were synthesized and evaluated. As shown in Figure 3A, hairpin probes with 8 and 9 base pairs in the stem (H8 and H9) were almost completely digested by DSN even in the absence of miR-199a-5p (lanes 6 and 8). Probes with shorter stems (H6 and H7) also exhibited partial cleavage (lanes 2 and 4). To further investigate the performance of H6, DSN cleavage was tested at two temperatures, 37 and 60 °C (Figure 3B). In the presence of miR-199a-5p, H6 was consistently cleaved at both temperatures (lanes 6 and 12). However, in the absence of the target, temperature-dependent stability was observed: partial cleavage occurred at 37 °C (lanes 4 and 5), whereas no obvious digestion was detected at 60 °C (lanes 10 and 11). This phenomenon may be attributed to temperature-induced conformational changes in the loop region, as supported by the predicted minimum free energy (MFE) structure of H6 from NUPACK analysis (Figure S3A). To avoid structural instability that may occur with fewer than five base pairs in the hairpin stem, a minimum of six base pairs was maintained in this region. On this basis, the loop sequence was adjusted based on the target-complementary region. To achieve optimal secondary structure formation at 37 °C, hairpin probes were further designed with varying loop sizes and differing numbers of target-complementary bases incorporated into the stem region, as detailed in Table S3. Figure 3C shows that, in the absence of the target, H1 and H3—both possessing larger loop sizes—exhibited greater resistance to DSN cleavage compared to H5. This stability is likely attributed to the spatial separation of the loop duplex structure from the stem region.

**Figure 3:**
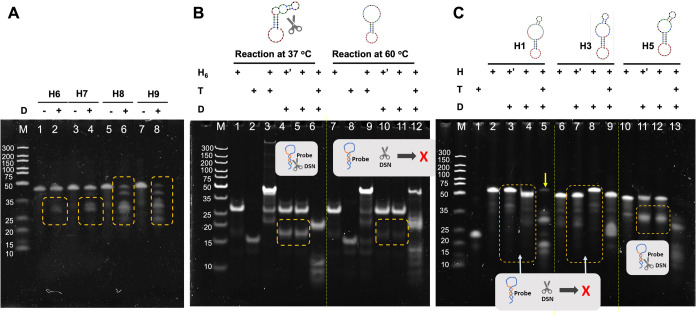
Characterization of the hairpin probe in the DSN reaction analyzed by denaturing PAGE (15%). (A) Hairpin probes with 6, 7, 8, and 9 base pairs in the stem were reacted with DSN in the absence of target miR-199a-5p; (B) comparison of DSN cleavage of hairpin probe H6 at 37 and 60 °C in the presence and absence of target miR-199a-5p; (C) hairpin probes with 22, 25, and 29 nucleotides in the loop region were reacted with DSN in the presence and absence of target miR-199a-5p. Denaturing PAGE: 15% acrylamide gel containing 7 M urea; running buffer: 0.5× TBE; electrophoresis conditions: 70 V, 85 min, 50 °C. M: marker; H: hairpin probe; T: target miR-199a-5p; + ’: group subjected to heating and slow annealing to ensure proper hairpin formation.

However, upon the introduction of target miR-199a-5p, the H1/miR-199a-5p duplex demonstrated enhanced DSN cleavage efficiency over H3/miR-199a-5p, suggesting that the number of target-complementary bases incorporated into the stem influences the efficiency of hairpin unfolding upon target binding. As indicated in Figure S3B,C, the melting temperature of H1 increased from 60.3 °C in the absence of the target to 71.2 °C after duplex formation with DNA-199a-5p. The melting analysis also verified that the “balloon-like” hairpin configuration—with a 29-nucleotide loop and a 6-base-pair stem—remained stably folded as a hairpin structure under the enzyme reaction conditions (37 °C). These findings confirm that this design functioned as intended and was therefore selected as the optimal hairpin DNA probe (hDNA) for subsequent experiments.

Further assessments revealed that, in the absence of target miR-199a-5p, the hDNA probe retained its structural integrity in the presence of DSN, as shown in Figure S4A (lane 7). Notably, DSN-mediated digestion of hDNA occurred only upon hybridization with miR-199a-5p (lane 8), indicating high specificity toward the target over other miRNAs commonly present in serum. In addition, comparative analyses with linear probes confirmed that the hairpin configuration conferred superior resistance to DSN cleavage, even in the presence of one, two, or three mismatches (Figure S4B), consistent with previous reports describing similar observations.^53^ Collectively, these results highlight the designed hairpin probe’s excellent specificity and structural stability, validating its suitability for precise miR-199a-5p detection. Importantly, the optimization process reveals critical probe design principles for DSN-compatible hairpin structures, establishing a practical and transferable framework that can be readily applied to the rational design of probes for other miRNA targets in future assays.

From this systematic evaluation, several key design principles can be derived: (1) a moderately short yet stable stem (∼6 base pairs) is required to suppress background DSN cleavage while permitting efficient target-induced unfolding; (2) an enlarged loop region spatially separates the duplex-forming segment from the stem, thus reducing unintended enzymatic accessibility and enhancing target binding; and (3) proper placement of target-complementary sequences ensures efficient duplex formation and effective DSN recognition upon hybridization. Together, these features define a “balloon-like” probe architecture that balances structural stability and amplification efficiency.

### Characterization of the hDNA-Conjugated Magnetic Beads (MB-hDNA)

The conjugation process of hDNA onto magnetic beads (MB-hDNA) was characterized using dynamic light scattering (DLS), fluorescence spectroscopy, and polyacrylamide gel electrophoresis (PAGE). Following modification with hDNA, a notable increase in hydrodynamic diameter was observed, as indicated in Figure S5A. In addition, the zeta potential of the MB shifted toward a more negative value (Figure S5B and Table S4), confirming successful conjugation of hDNA onto the bead surface.

To further verify successful conjugation, the supernatant from the MB-hDNA conjugation solution was analyzed after staining with SYBR Gold fluorescent dye. For the MB–hDNA conjugates, the probe density on each magnetic bead was estimated to be approximately 4,048 strands, as calculated from the fluorescence quantification of the SYBR Gold signal (Figure S6 and Table S5). Subsequently, aliquots of miR-199a-5p were added to the MB–hDNA solution, followed by the collection of the supernatant for gel electrophoretic analysis. The PAGE results shown in Figure S5C (lane 6) demonstrated the absence of residual target strands or probes in the supernatant, confirming the stable attachment of hDNA to the magnetic beads and the effective hybridization capability of the MB–hDNA conjugates with the target. Moreover, the MB–hDNA/target complexes could be readily dissociated and released into the solution by simple heat treatment at 95 °C, as evidenced in lane 7 of the exact figure. This observation highlights the dynamic hybridization behavior of the magnetic bead–based system.

### Characterization and Optimization of the Synthesis Conditions of CuNCs

To further validate the feasibility of DNA-templated copper nanocluster (CuNC) formation, polyT DNA was synthesized and employed as a template for CuNC preparation. The optical properties of the resulting CuNCs, including their absorption, excitation, and emission spectra, are presented in Figure S7A,B. Notably, the DNA–CuNCs spectrum exhibited a subtle shoulder around 340 nm that was not observed in the pure DNA sample, which may correspond to a characteristic absorption feature of the Cu nanoclusters.

To further optimize the synthesis conditions for the polyT DNA–templated copper nanoclusters (CuNCs), we selected copper sulfate as the primary copper ion source, as commonly reported in previous studies.^54^ In addition, both sodium ascorbate and ascorbic acid were examined as reducing agents.^55^ Furthermore, three different copper ion sources—copper sulfate, copper carbonate, and copper nitrate—were compared in combination with these two reducing agents to evaluate their effects on CuNC formation. As shown in Figure S8A,B, CuNCs synthesized using copper sulfate exhibited markedly higher fluorescence intensity than those prepared with copper carbonate or copper nitrate. In contrast, only minor differences were observed between reactions employing sodium ascorbate (SA) and ascorbic acid (AA) as reducing agents. Based on these results, copper sulfate and sodium ascorbate were selected as the optimal copper ion source and reducing agent, respectively, for subsequent experiments. The optimized concentrations were determined to be 200 μM for Cu^2+^ (Figure S8C,D) and 2 mM for sodium ascorbate (Figure S8E,F). We first evaluated whether common cations present in the enzymatic reaction buffer would interfere with the fluorescence of DNA-templated copper nanoclusters. As shown in Figure S9A,B, the presence of K^+^ (50 mM), Mg^2+^ (10 mM), and Co^2+^ (0.25 mM) did not cause any significant change in the fluorescence intensity of the polyT–CuNCs, indicating good tolerance toward these metal ions under our reaction conditions. We next examined the buffer composition on CuNC fluorescence by comparing MOPS buffer, TdT reaction buffer, and a mixture of the two. Although the fluorescence intensity of polyT–CuNCs was slightly reduced in TdT buffer relative to MOPS buffer alone, the addition of 10 mM MOPS to the TdT reaction buffer effectively preserved the fluorescence signal (Figure S9C,D). Because previous studies have reported that polyT-templated CuNCs may exhibit limited stability due to oxidation-induced fluorescence quenching,^56^ we further evaluated the temporal stability of CuNC fluorescence under optimized reaction buffer conditions. Fluorescence spectra were recorded after 2.5, 5, 10, 15, and 30 min of incubation (Figure S10A). As summarized in Figure S10B, the fluorescence signal remained relatively stable over the 30 min monitoring period, with only minor signal decay observed. Based on this stability profile, an incubation time of 5 min was selected for subsequent experiments to balance signal robustness and assay throughput.

### Feasibility Investigation of the Biosensing Platform

The hybridization of target miR-199a-5p with MB–hDNA effectively initiated DSN-mediated digestion of the hDNA probe, as shown in Figure S11A. The MB–hDNA/miR-199a-5p complexes were completely cleaved by 0.5 U DSN within 30 min (lane 8). Remarkably, DSN exhibited enhanced digestion efficiency toward the hDNA/miR-199a-5p duplex when immobilized on magnetic beads, compared with the digestion of free hDNA in solution (lane 3), even at a higher DSN concentration (lane 4). This enhancement is likely attributable to the spatial proximity and favorable orientation provided by the bead surface, which may facilitate more efficient enzyme–substrate (hDNA/miR-199a-5p duplex) interactions.

The DSN-assisted amplification process follows a cyclic mechanism involving (1) hybridization between target miRNA and immobilized hairpin probes, (2) DSN-mediated cleavage of the DNA strand within the DNA–RNA duplex, and (3) release of intact miRNA to initiate subsequent cycles. Although surface immobilization may introduce steric hindrance and slightly reduce hybridization efficiency, this effect is not dominant under our experimental conditions. Instead, the high local probe concentration facilitates efficient duplex formation and supports enzymatic turnover. Notably, no significant reduction in DSN or TdT activity was observed, indicating that enzyme accessibility remains largely preserved. Overall, the amplification efficiency reflects a balance between steric constraints and localized concentration effects.

Following the DSN-mediated reaction, as shown in Figure S11B, MB-hDNA without subsequent elongation by TdT and dTTP failed to template CuNC formation, resulting in minimal fluorescence (curve a). In the absence of miR-199a-5p, the 3′ terminally phosphorylated hDNA was neither cleaved by DSN nor extended by TdT, producing only weak background fluorescence (curve b). Conversely, the presence of target miR-199a-5p triggered the generation of fluorescent CuNCs, characterized by a strong emission peak upon excitation at 340 nm (curve c). The morphology of bare MBs and MB–CuNCs was further examined by transmission electron microscopy (Figure S11C), confirming the successful formation of CuNCs on the bead surface. We observed that only a limited number of Cu nanoclusters were present on the bead surface, reflecting the combined effects of the multistep amplification process. At the selected reaction time, only a fraction of immobilized probes undergoes target hybridization, DSN-mediated cleavage, and subsequent TdT-mediated polyT elongation, thus limiting the number of available nucleation sites. In addition, PAGE analysis (Figure S12) indicates that TdT generates polyT strands with heterogeneous lengths, and only sufficiently elongated strands are capable of supporting efficient Cu nanocluster formation. As CuNC nucleation is highly dependent on polyT length, the efficiency of TdT-mediated elongation plays an imporant role in determining the final distribution of CuNCs. These factors collectively result in a nonuniform CuNC distribution on the bead surface, consistent with the heterogeneous nature of surface-confined enzymatic reaction.^57^ Importantly, despite this heterogeneity, the optimized reaction conditions provide reproducible fluorescence signals for analytical detection (Figure S11B), indicating that sensing performance depends on the consistent generation of sufficient signal-producing sites rather than the absolute number of Cu nanoclusters.

### Assay Optimization

After establishing the feasibility of our proposed assay design, we proceeded to systematically investigate and optimize key experimental parameters to improve its overall performance. Specifically, we examined the optimal quantity of MB-DNA required for the effective TdT reaction. To this end, we first utilized a linear biotinylated DNA probe (Pcut) bearing a free 3′-terminal hydroxyl group, which enabled direct recognition by TdT and thus eliminated the requirement for prior DSN digestion. As revealed in Figure S13A 20 μL MB-DNA solutions was used for further experiments. To further evaluate the effect of probe density on DSN reaction efficiency, hairpin probes were immobilized on magnetic beads at two different densities and analyzed by PAGE (Figure S14). The results indicate that probe density has a limited effect on DSN cleavage efficiency under the investigated conditions. Although higher probe density may introduce steric hindrance and partially restrict target accessibility, the localized high-density microenvironment can compensate by increasing the effective concentration of DNA–RNA duplex substrates and facilitating catalytic turnover. Consistently, the data in Figure S13B show that probe density also has minimal impact on TdT-mediated extension efficiency within the tested range. These observations suggest that steric hindrance and localized concentration effects exert competing influences, resulting in an overall balanced system. Among the conditions examined, a probe density of ∼4048 strands per bead yielded slightly higher fluorescence signals and was therefore selected for subsequent experiments.

To further refine the assay conditions, we optimized the TdT-mediated polymerization step using MB–hDNA. As demonstrated in Figure S13C, the optimal reaction duration following DSN digestion was approximately 1.5 h. Compared with the typical TdT reaction time of 2–3 h,^58^ the enhanced extension efficiency was likely attributed to the surface-induced enzymatic proximity (SIEP) effect on MB–hDNA.^57^ This behavior is consistent with results obtained in free solution (Figure S12), where PAGE analysis showed a time-dependent shift toward higher molecular weight products (0.5–4.5 h), indicating progressive polyT elongation. The smear pattern reflects the heterogeneous nature of TdT-mediated extension. Notably, partial extension was observed at shorter reaction times (e.g., 1.5 h), while more extensive elongation occurred at longer durations. Although PAGE does not provide absolute quantification of polyT length or yield, it offers direct evidence of TdT activity and enables a semiquantitative evaluation of extension efficiency. Together with the fluorescence results, these findings support the existence of an optimal reaction time that balances elongation efficiency and overall signal output.

Subsequently, we investigated the optimal concentrations of dTTP and TdT. Our findings, illustrated in Figure S13D,E, revealed that a dTTP concentration of 4 mM combined with 20 units of TdT produced the most pronounced fluorescence signals. Building on these results, we further refined the probe design by examining the nucleotide sequence serving as the “linker” region within the hDNA structure. Linkers composed of 4, 8, 12, 16, and 20 thymidine bases were synthesized and evaluated. As shown in Figure S13F, the hDNA containing an 8-thymidine linker exhibited the most effective performance. To elucidate the underlying reason, we investigated how different linker lengths influence DSN digestion efficiency on the MB–hDNA/target duplex, as characterized in Figure S15. The results suggested that linker length plays an important role in modulating the accessibility and catalytic efficiency of DSN, with the 8-base linker optimally facilitating the reaction—in consistence with previous reports.^59,60^ This behavior can be attributed to spatial effects at the bead surface. The magnetic beads (∼100 nm), coated with streptavidin, are functionalized with biotinylated probes, where variations in polyT linker length modulate the distance between the probe and the bead surface. Even at comparable probe densities, differences in linker length affect probe orientation and accessibility, thus influencing both hybridization and enzymatic reactions. These results highlight that linker length is a key parameter in balancing steric hindrance and local concentration effects, ultimately defining an optimal microenvironment for efficient DSN cleavage and TdT-mediated amplification.

To further assess the robustness of the optimized assay conditions, we examined both intra- and interday reproducibility of the proposed sensing platform over three consecutive testing days (Figure S16). For each day (Tests 1–3), triplicate measurements performed at the same target concentration yielded intraday coefficients of variation (CVs) of 7.90%, 1.34%, and 2.16%, respectively, indicating good precision within individual assay runs. To examine day-to-day consistency, the average fluorescence intensities obtained on the three separate days were compared, resulting in an interday CV of 4.18%. Collectively, these results demonstrate that the developed sensing platform exhibits reliable reproducibility both within single assay runs and across multiple testing days.

### Assay Performance

To evaluate the analytical utilities of our developed assay, we studied its sensitivity and selectivity under the optimized experimental conditions. As shown in Figure 4A,B, significant fluorescence was observed exclusively in the presence of miR-199a-5p and in the pooled sample containing miR-199a-5p together with other common serum miRNAs related with gynecological diseases (miR-141, miR-21, and let-7a).^13,14,61^ In contrast, negligible fluorescence signals were detected when only nontarget miRNAs were introduced, demonstrating the system’s excellent selectivity. The signal-to-noise (S/N) ratio for miR-199a-5p was markedly higher than that of the other tested miRNAs, confirming that the DSN-assisted amplification process is strictly dependent on the formation of the miR-199a-5p/hDNA duplex. These results collectively demonstrate the high specificity and analytical reliability of the proposed biosensing platform for detecting miR-199a-5p.

**Figure 4:**
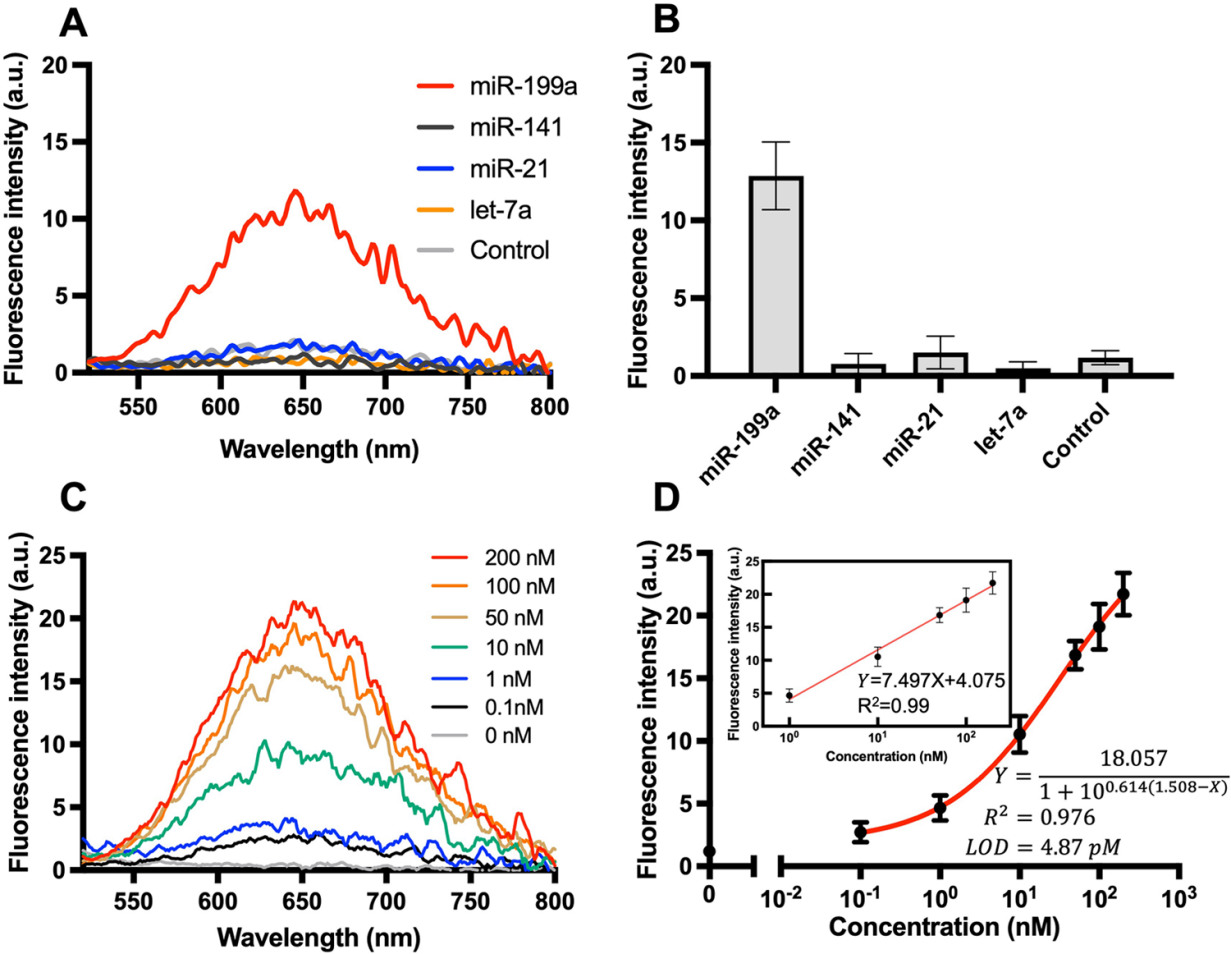
Analytical performance of the DSN-TdT dual-mode MB-hDNA assay for miR-199a-5p detection. (A) Representative fluorescence spectra obtained using various miRNA targets, including miR-199a-5p, miR-141, miR-21, and let-7a. (B) Selectivity evaluation of the assay based on the maximum fluorescence intensities of the samples in (A), recorded under 340 nm excitation. (C) Representative fluorescence spectra obtained upon the addition of different concentrations of miR-199a-5p (0, 0.1, 1, 10, 50, 100, and 200 nM); (D) calibration curve of miR-199a-5p detection, plotted as fluorescence peak intensity versus miRNA concentration. The limit of detection (LOD) was calculated to be 4.87 pM, using the 3σ criterion. Error bars represent the standard deviation from three independent measurements. All fluorescence spectra were recorded at an excitation wavelength of 340 nm (λ_ex_ = 340 nm) and scanned from 500 to 800 nm, with maximum emission at 650 nm. Error bars represent the standard deviation of triplicate measurements.

The fluorescence spectra of CuNCs obtained in response to varying concentrations of miR-199a-5p (0.1 nM to 200 nM) are presented in Figure 4C. The relationship between miR-199a-5p concentration and the fluorescence intensity of CuNCs was fitted using a four-parameter sigmoidal model, yielding an excellent correlation coefficient (*R* ^2^ = 0.976). According to the IUPAC definition, the limit of detection (LOD), calculated as three times the standard deviation above the blank signal, was determined to be 4.87 pM (Figure 4D). In addition, a useful linear response region was observed over the concentration range of 1–200 nM (inset in Figure 4D). Overall, the DSN–TdT dual-mode assay combines high sensitivity, rapid analysis, and experimental simplicity, demonstrating performance comparable to or superior to that of previously reported miRNA biosensors. Although several DNA–CuNC- or DSN–TdT-based sensors have achieved excellent analytical sensitivity, they typically require longer assay times and are seldom validated using clinical samples (Table S6). In the present work, fluorescence signals were acquired using a standard microplate reader rather than a fluorescence spectrometer. While this choice may result in lower absolute signal intensities and thus a modest compromise in sensitivity, it enables parallel measurements in multiwell formats and substantially improves assay throughput. Notably, the use of a microplate reader enhances the practicality of the platform for high-throughput miRNA analysis, while further improvements in detection limits could be readily achieved by adopting advanced spectroscopic readout systems when higher sensitivity is required. Taken together, our proposed strategy offers a balanced combination of sensitivity, operational simplicity, assay speed, and real-sample applicability, making it well-suited for routine analysis and potential clinical translation.

### Detection of miR-199a-5p in Serum Samples Using the DSN-TdT Dual Mode MB-hDNA Assay

Finally, the clinical applicability of the proposed biosensing platform was assessed for quantifying miR-199a-5p in human serum, with particular emphasis on its ability to distinguish patients with endometriosis from healthy controls. To characterize matrix effects and verify assay performance in complex biological media, spike–recovery experiments were conducted using two matrices: fetal bovine serum (FBS) and healthy human serum. Synthetic miR-199a-5p (100 nM) was added and all specimens were processed identically to clinical samples. The percent recovery (%Recovery) was calculated as the ratio of the amount recovered to the initial amount added, multiplied by 100, according to the eq 2

2 $$\%\;\mathrm{recovery}=\frac{\mathrm{amount}\;\mathrm{recovered}}{\mathrm{amount}\;\mathrm{added}}\times 100\%$$

As shown in Figure S17, our biosensing platform demonstrated consistent detection performance across two tested sample types, with no statistically significant differences observed among matrices, confirming its compatibility with biological environments. Serum samples were collected and subsequently analyzed from two distinct groups: ten individuals clinically diagnosed with endometriosis (EM+) and ten healthy controls (EM−). After RNA extraction, as described in the Experimental Section, the concentration of miR-199a-5p was quantified using the proposed biosensing platform. The resulting scatter plots comparing miR-199a-5p levels obtained by our assay and a commercial TaqMan-based qRT-PCR kit are displayed in Figure 5, with supplementary comparisons provided in Figure S18A,B and Table S7.

**Figure 5:**
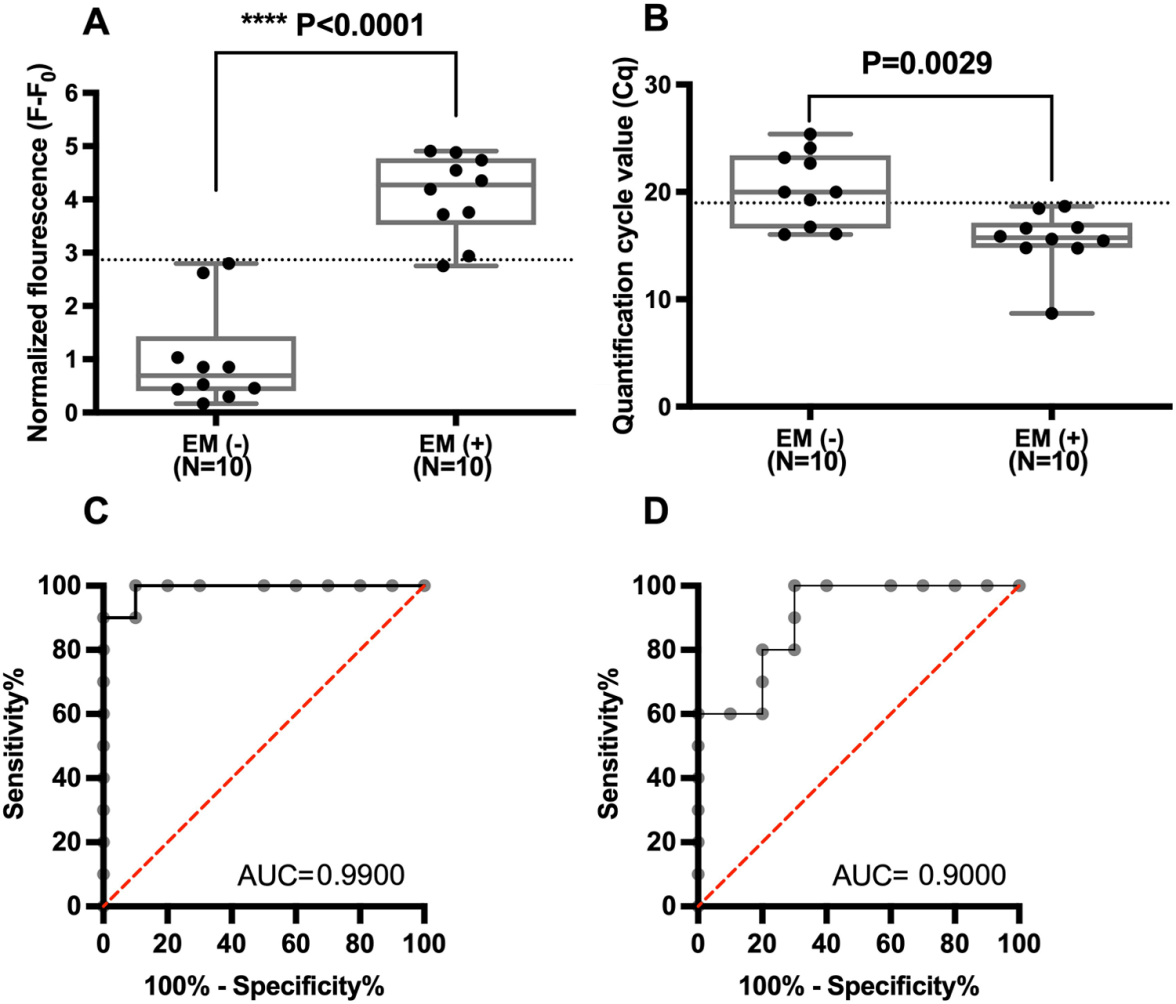
Scatter plots showing the analysis of serum samples from individuals with endometriosis (EM+, *N* = 10) and healthy controls (EM–, *N* = 10). (A) Serum samples were analyzed using the proposed biosensing platform. The optimal cutoff value (2.866) for maximizing sensitivity and specificity was derived from the ROC curve shown in (C); (B) serum samples were analyzed using a commercial miRNA qRT-PCR kit for comparison. The optimal cutoff value (18.97) for maximizing sensitivity and specificity was derived from the ROC curve shown in (D); (C) ROC curve constructed using results from the proposed biosensing platform. The AUC was 0.9900 (*p* < 0.001); (D) ROC curve constructed using results from the commercial miRNA qRT-PCR kit. The AUC was 0.9000 (*p* = 0.0025).

The results summarized in Figure 5 reveal several key findings. First, both analytical approaches—the proposed biosensing platform (Figure 5A) and conventional qRT-PCR (Figure 5B)—successfully distinguished miR-199a-5p levels between patients with endometriosis and healthy controls, further supporting the diagnostic relevance of miR-199a-5p. Receiver operating characteristic (ROC) curves were generated for the biosensing platform (Figure 5C) and qRT-PCR (Figure 5D), and cutoff values were selected by maximizing the combined sensitivity and specificity. In this cohort, the area under the curve (AUC) obtained with the biosensing platform (AUC = 0.9900) was slightly higher than that of qRT-PCR (AUC = 0.9000), indicating excellent diagnostic performance for both methods. In addition, two control samples (Nos. Twelve and 20) exhibited slightly elevated fluorescence signals in the proposed assay, accompanied by correspondingly lower Cq values in qRT-PCR analysis (Figure S18). This deviation may be attributable to the presence of adenomyosis in these individuals, which has been reported to influence circulating miRNA profiles and could partially account for the observed signal variation. Taken together, these results demonstrate that the proposed biosensing platform provides robust analytical accuracy and good reproducibility for quantifying miR-199a-5p in complex biological matrices, highlighting its potential as a reliable tool for precise miRNA detection and clinical discrimination of endometriosis. We acknowledge that the present study is limited by sample size, which primarily reflects the practical challenges associated with the collection of well-characterized clinical specimens. While our results indicate that miR-199a-5p holds promise as a diagnostic biomarker for endometriosis, further validation in larger and more diverse patient cohorts is clearly warranted. In future work, we plan to expand the sample size to encompass patients across different disease stages, histopathological subtypes, and molecular classifications of endometriosis.^14,62–64^ Longitudinal analyses will also be conducted to monitor miR-199a-5p levels before and after treatment, correlate expression dynamics with therapeutic response, and assess its potential association with disease recurrence during follow-up. Such efforts will enable a more comprehensive assessment of the biosensor not only as a screening tool but also as a potential prognostic and disease-monitoring platform. Importantly, the value of the present work lies not solely in the evaluation of a single biomarker, but in the establishment of a flexible and modular assay framework. Based on the prescreening results (Figure 1D), the platform can be readily adapted to other endometriosis-associated miRNAs, such as miR-143–3p and miR-145–3p, through simple probe redesign while preserving the same amplification and fluorescence readout scheme. This adaptability positions the proposed method as a broadly applicable platform for future multiplexed biomarker analysis in endometriosis and other gynecological diseases.

## Conclusion

In this study, we integrated bioinformatic prescreening with a CuNC-based biosensing platform to enable rational miRNA target selection and sensitive detection. Differential expression analysis of publicly available miRNA-sequencing data sets was first used to prioritize candidate biomarkers for endometriosis, from which miR-199a-5p was selected as a representative target based on its consistent dysregulation and reported diagnostic relevance. Building on this, we established a novel, sensitive, and highly specific biosensing platform for miR-199a-5p detection, integrating a rationally engineered hairpin DNA probe with duplex-specific nuclease (DSN)-assisted signal amplification, which operates under physiological conditions (37 °C). By exploiting the surface-initiated enzymatic polymerization (SIEP) effect on magnetic beads, our design significantly enhanced enzymatic efficiency and reduced reaction time compared to conventional homogeneous systems, allowing the entire analytical process to be completed within 2 h. The platform exhibited exceptional specificity, effectively discriminating miR-199a-5p from closely related sequences containing one to three mismatches, and achieved a detection limit of 4.87 pM. To improve assay throughput and operational feasibility, we incorporated an automated magnetic separation module into the workflow. The resulting measurements were statistically consistent with those obtained by manual handling, while offering the additional benefits of reduced protein-related interference and improved control over reaction conditions.

Importantly, the clinical performance of the proposed biosensing platform was further substantiated through direct comparison with conventional qRT-PCR analysis using patient serum samples. Both methods successfully discriminated patients with endometriosis from healthy controls based on circulating miR-199a-5p levels, confirming its diagnostic relevance. Notably, the receiver operating characteristic (ROC) analysis revealed that the biosensing platform achieved an area under the curve (AUC) of 0.9900, which was slightly higher than that obtained by qRT-PCR (AUC = 0.9000), indicating excellent diagnostic accuracy for both approaches. These results demonstrate that the proposed assay can achieve clinical performance comparable to the current gold standard while operating under simpler and faster experimental conditions. A detailed side-by-side comparison of workflow complexity, assay duration, reagent cost, and instrumentation requirements between the two methods is provided in Table S8. Looking ahead, the platform is inherently compatible with automated magnetic separation systems, which may further streamline miRNA extraction, minimize manual sample-processing steps, and simplify the overall workflow for routine clinical implementation.^65^ While larger clinical cohorts will be required for comprehensive validation, the current results establish a solid proof of concept for translating chemically engineered, amplification-efficient miRNA sensing into clinically meaningful analysis. Overall, this study demonstrates that chemically programmed, amplification-efficient miRNA biosensing can be translated into a clinically relevant analytical platform, offering a practical balance between sensitivity, robustness, and operational simplicity for endometriosis-related diagnostics.

## Supplementary Material



## Notes

Listing the sequences of oligonucleotides (Table S1) and design (Table S3) used in this study, detection workflow summary (Table S2), comparison of dynamic light scattering results for MB-hDNA and bare MB (Table S4), calculation of probe density (Table S5), comparison of various biosensor platforms (Table S6), clinical characteristics of patients with endometriosis or without endometriosis (Table S7) and comparison between the proposed biosensor and the clinical standard method (Table S8); supplementary figures demonstrating heatmap of differentially expressed miRNAs in two data sets (Figure S1 and S2), rational design of probes (Figure S3) and characterization (Figure S4), characterization of MB-hDNA (Figure S5 and S6), characterization of fluorescent polyT-templated CuNCs (Figure S7), optimization of the synthesis conditions for polyT-templated CuNCs (Figure S8), the effects of reaction buffer composition on the property of polyT-templated CuNCs (Figure S9), fluorescence stability of polyT-templated CuNCs (Figure S10), feasibility assessment of the biosensing platform (Figure S11), characterization of TdT-mediated extension in free solution at different reaction times (Figure S12), assay optimization (Figure S13), characterization of hairpin probes immobilized at different probe density (Figure S14) and containing different thymidine linkers (Figure S15) in the DSN-assisted amplification reaction on magnetic beads, intra-and inter- day reproducibility of the dual mode MB-hDNA assay (Figure S16), evaluation of matrix effects using a spiked-in sample in different biological media (Figure S17), and quantitative analyses of the. serum samples from individuals with endometriosis and healthy controls (Figure S18) (PDF)

## ○

Department of Chemistry, University of Wisconsin-Madison, 1101 University Avenue, Madison, Wisconsin 53706, United States
