# Supplementary material for: Diagnosis of Endometriosis: Dual-Amplification Strategy Driven by Copper Nanoclusters
Source: Anal Chem. 2026 May 14;98(20):15002–15. doi: 10.1021/acs.analchem.6c00532 (PMC13217368; doi:10.1021/acs.analchem.6c00532)
Supplement: Supplementary file 1 [file ac6c00532_si_001.pdf]

Supporting Information

Diagnosis of Endometriosis: Dual-Amplification Strategy Driven by Copper Nanoclusters

Yu-Ling Wu[a], Hsu-Ching Yen[aϕ], Pao-Ling Torng[b,c*], and Ja-an Annie Ho[a,d,e,f,g*]

[a]BioAnalytical Chemistry and Nanobiomedicine Laboratory, Department of Biochemical Science and Technology, National Taiwan University, 106319 Taipei, Taiwan

[b]Department of Obstetrics and Gynecology, National Taiwan University Hospital, 100217 Taipei, Taiwan

[c]Department of Obstetrics and Gynecology, Tungs' Taichung MetroHarbor Hospital, 435403 Taichung, Taiwan

[d]Department of Chemistry, National Taiwan University, 106319 Taipei, Taiwan

[e]Center for Emerging Materials and Advanced Devices, National Taiwan University, 106319 Taipei, Taiwan

[f]Center for Biotechnology, National Taiwan University, 106319 Taipei, Taiwan

[g]Professional Master's Program of Biotechnology Management, School of Professional Education and Continuing Studies, National Taiwan University, 106319 Taipei, Taiwan

[ϕ] Hsu-Ching Yen is currently at the Department of Chemistry, University of Wisconsin-Madison, 1101 University Avenue, Madison, Wisconsin 53706, USA

[*]Corresponding Authors. Pao-Ling Torng: pltorng0827@gmail.com; Ja-an Annie Ho: jaho@ntu.edu.tw

Table of Contents

Supplemental Tables

Table S1. ……..………..…………………………….…………………………………...S4

Table S2. ……..………..…………………………….…………………………………...S5

Table S3. ……..………..…………………………….…………………………………...S6

Table S4. ……..………..…………………………….…………………………………...S6

Table S5. ……..………..…………………………….…………………………………...S7

Table S6. ……..………..…………………………….…………………………………...S8

Table S7. ……..………..…………………………….…………………………………......S9

Table S8. ……..………..…………………………….………………………………….....S10

Supplemental Figures

Figure S1. …….………...……………………….……………………………S11

Figure S2. ……….……………………………………………………………….S12

Figure S3. ……………………………………………………………………..S13

Figure S4. ……………………………………………………………………..S14

Figure S5. ……………………………………….…..…………………………..S15

Figure S6. ……………………………………….…..…………………………..S16

Figure S7. ……………………………………………………………………..S17

Figure S8. ……………………………………….…..…………………………..S18

Figur.S9. ……………………………………….…..…………………………...S19

Figure S10. ……………………………………….…...………………..……..S20

Figure S11. ……………………………………….…...………………..……..S21

Figure S12. ……………………………………….…...………………..……..S22

Figure S13. ……………………………………….…...………………..……..S23

Figure S14. ……………………………………….…...………………..……..S24

Figure S15. ……………………………………….…...………………..……..S25

Figure S16. ……………………………………….…...………………..……..S26

Figure S17. ……………………………………….…...………………..……..S27

Figure S18. ……………………………………….…...………………..……..S28

Table S1. The sequences of the oligonucleotides used in this study.

Name	Sequence (5’-3’)
H1	Biotin- TTTTTTTTTTTTCCCAGCATTTTTAGAACAGGTAGTCTGAACACTGGGCTGGG-PO_4
H3	TTTTTTTTTTTTAAGCCCCCCAGAACAGGTAGTCTGAACACTGGGCTT
H5	TTTTTTTTTTTTACCCAGGTGAGAACAGGTAGTCTGAACACTGGGT
H6	TTTTTTTTTTTTCCCAGTTGAGAACAGGTAGTCTGAACACTGGG
H7	TTTTTTTTTTTTCCCAGTTGAGAACAGGTAGTCTGAACACTGGGA
H8	TTTTTTTTTTTTCCCAGTGTGAGAACAGGTAGTCTGAACACTGGGA
H9	TTTTTTTTTTTTCCCAGTGTGAGAACAGGTAGTCTGAACACTGGGAA
H1-4T linker	Biotin-TTTTCCCAGCATTTTTAGAACAGGTAGTCTGAACACTGGGCTGGG-PO_4
H1-8T linker	Biotin-TTTTTTTTCCCAGCATTTTTAGAACAGGTAGTCTGAACACTGGGCTGGG-PO_4
H1-16T linker	Biotin-TTTTTTTTTTTTTTTTCCCAGCATTTTTAGAACAGGTAGTCTGAACACTGGGCTGGG-PO_4
H1-20T linker	Biotin-TTTTTTTTTTTTTTTTTTTTCCCAGCATTTTTAGAACAGGTAGTCTGAACACTGGGCTGGG-PO_4
Pcut	Biotin-TTTTTTTTTTTTTT
DNA-199a-5p	CCCAGTGTTCAGACTACCTGTTC
miR-199a-5p	CCCAGUGUUCAGACUACCUGUUC
T60	TTTTTTTTTTTTTTTTTTTTTTTTTTTTTTTTTTTTTTTTTTTTTTTTTTTTTTTTTTTT
miR-21	UGAGGUAGUAGGUUGUAUAGUU
Let-7a	UAGCUUAUCAGACUGAUGUUGA
miR-141	UAACACUGUCUGGUAAAGAUGG
miR-200a	UAACACUGUCUGGUAACGAUGU
DNA 199a 1- mutate	CCAAGTGTTCAGACTACCTGTTC
DNA 199a 2- mutate	CCAAGTGTTGAGACTACCTGTTC
DNA 199a 3- mutate	CCAAGTGTTGAGACTACATGTTC

Table S2. Summary of the overall detection workflow and corresponding time requirements of the developed biosensing platform.

Step		Materials	Time
**Preparation of Materials**	Extraction of miRNA from serum samples	miRNA extraction kits<br>Serum sample	1 h
	MB-hDNA	5'-biotinylated and 3'-phosphorylated DNA oligonucleotides, Streptavidin-coated magnetic bead	30 min
	Magnetic separation	PBS buffer, magnet	5 min
**Detection of Targets**	Target and DSN reaction	Target solution, DSN, DSN reaction buffer	30 min
	Magnetic separation	PBS buffer	5 min
	TdT-mediated extension reaction	TdT, dTTP, TdT reaction buffer	90 min
	Copper nanoclusters synthesis	$CuSO_4$, sodium ascorbate, MOPS buffer	5 min

Table S3. Hairpin sequences with varying loop sizes and different numbers of target-complementary bases in the stem region.

Name	Stem length (bp)	Loop size (nt)	Complementary nucleotides in the stem (nt)
H1	6	29	5
H3	6	24	3
H5	6	22	1

Table S4. Comparison of dynamic light scattering (DLS) results for MB–hDNA and bare MB. Z-average represents the intensity-weighted harmonic mean hydrodynamic diameter, and PDI denotes the polydispersity index obtained from DLS measurements

Sample	Z-average (nm)	PDI	Zeta potential (mV)
SA-MB	174.4±1.5	0.10±0.06	-21.6±0.5
SA-MB-hDNA	191.5 ± 3.1	0.10±0.10	-34.1±1.8

Table S5. Calculation of probe density (DNA amount per magnetic bead). The standard deviation represents the results of three independent experiments.

	Average	Standard deviation
Fluorescence Intensity (a.u.)	23.49	2.16
Conc. (nM) (in supernatant)	6.89	0.65
Conc. (nM) (on MB)	186.23	1.30
~hDNA on MB (pmol)	9.31	0.06

The amount of DNA immobilized on 10 μL MB is calculated using the **Equation S1** from:

$$\text{DNA amount (pmol)} = \Delta conc.\ (nM) \times 10^{-9} \times \text{volume}\ (\mu L) \times 10^{-6} \times 10^{12} \quad \text{S1}$$

The strands of DNA immobilized per MB is calculated using the **Equation S2** from:

$$\text{DNA strands/MB} = \text{DNA amount (pmol)} \times \text{volume}\ (\mu L) \times 1.38 \times 10^{8} \quad \text{S2}$$

Table S6. Comparison of various biosensing platforms for miRNAs detection with the developed sensing platform in this study

Biosensor Type	Sample Type	Target MiRNA(s)	Amplification/Signal Strategy	LOD	Assay Time	Instrument	Reference
Electrochemical biosensor	Spike assay	miR-21	DNA-templated Cu nanoclusters (DNA-CuNCs) with [a]HCR + Exonuclease T7	10 aM	>4.5 h	Voltammetry	1
Electrochemical biosensor	Spike assay	miR-141	DSN, HCR, DNA and ratiometric assay (thiol-modified and ferrocene-labeled hairpin)	11 aM	2.5 h	Voltammetry	2
Electrochemical biosensor	Cell lysate	miR-21	DNA-templated Cu nanoclusters with toehold-mediated strand displacement, DNAzyme and TdT reaction	36 aM	>3 h	Voltammetry	3
[b]ECL biosensor	Spike assay	miR-155	DNA-templated Cu nanoclusters with binding-induced DNA assembly on a tetrahedral DNA to generate AT-rich sequences	36 aM	>1.5 h	Electrochemi-luminescence analyzer	4
Optical biosensor	Cell lysate	miR-21 (breast cancer)	DSN/TdT cyclic amplification (probe labeled with fluorophore and quencher)	5.4 pM	4.5 h	Fluorescence spectrometer	5
Optical biosensor	-	miR-21, miR-155 and miR-195 (breast cancer)	DNA-templated Cu nanoclusters (DNA-CuNCs) with HCR	1.7 pM	~2.5 h	Fluorescence spectrometer	6
Optical biosensor	Cell lysate	miR-21	DNA-templated Cu nanoclusters (DNA-CuNCs) with DSN and TdT dual amplification strategy	18.7 pM	>8 h	Fluorescence spectrometer	7
Optical biosensor	Spike assay	miR-155	DNA-templated Cu nanoclusters (DNA-CuNCs)	2.2 pM	>12 h	Luminescence spectrometer	8
Optical biosensor	Serum sample	miR-199a (endometriosis)	DNA-templated Cu nanoclusters (DNA-CuNCs) with DSN and TdT dual amplification strategy on magnetic beads	4.9 pM	~2.5 h	Microplate reader	This work

Note: [a]HCR stands for hybridization chain reaction and [b]ECL stands for electrochemiluminescence.

Table S7. Clinical characteristics of patients with endometriosis or without endometriosis.

Sample	Clinical diagnosis	Age (year)	Tumor size (cm)	CA-125 (U/mL)
**With endometriosis**				
**1**	Unilateral ovarian endometrioma	28	10x8	115.7
**2**	Unilateral ovarian endometrioma	35	16x11	36.1
**3**	Bilateral ovarian endometrioma, Adenomyosis	36	Right: 4x4<br>Left: 8x8	143.4
**4**	Unilateral ovarian endometrioma	33	8x6	-
**5**	Bilateral ovarian endometrioma, Adenomyosis	48	Right: 5x6<br>Left: 4x4	110
**6**	Unilateral ovarian endometrioma, Adenomyosis	42	10x8	-
**7**	Bilateral ovarian endometrioma	32	Right: 5x5<br>Left: 8x5	-
**8**	Bilateral ovarian endometrioma	39	Right: 5x4<br>Left: 6x5	-
**9**	Adenomyosis	49	-	-
**10**	Unilateral ovarian endometrioma	26	7x5	-
**Without endometriosis**				
**11**	Ovarian teratoma	32	6x5	13.7
**12**	Ovarian teratoma	33	9x8	-
**13**	Ovarian teratoma	30	5x4	30.8
**14**	Ovarian cancer (endometrioid adenocarcinoma)	44	10x10	-
**15**	Ovarian mucinous cystadenoma	39	7x6	-
**16**	Ovarian teratoma	35	6x6	46.3
**17**	Ovarian cancer (clear cell carcinoma)	60	16x15	1656
**18**	Ovarian cancer (clear cell carcinoma)	40	12x12	76.1
**19**	Ovarian Seromucous cystadenoma	32	9x8	-
**20**	Recurrent Ovarian teratoma with severe adhesion	40	7x6	-

Table S8. Comparison between the developed DSN–TdT dual-mode MB–hDNA assay and the clinical gold-standard qRT-PCR method.

	This Assay		qRT-PCT	
	**Procedure**	**Time**	**Procedure**	**Time**
**Sample Preparation**	Extraction of miRNA from serum samples	~1 h	Extraction of miRNA from serum samples	~ 1 h
	**Procedure**	**Time**	**Procedure**	**Time**
**Detection of Targets**	Target and DSN reaction	30 min	Reverse transcription	~ 1 h
	TdT-mediated extension reaction	90 min	PCR reaction	~ 1 h
	Copper nanoclusters synthesis	5 min		
**Total Assay Time**	~ 2 h		~ 2 h	
**Total Cost (per sample)**	~ 4.6 USD for testing materials/assay<br>microplate reader required		~ 5.6 USD for testing materials/assay<br>qPCR machine required<br>+ trained personnel required	

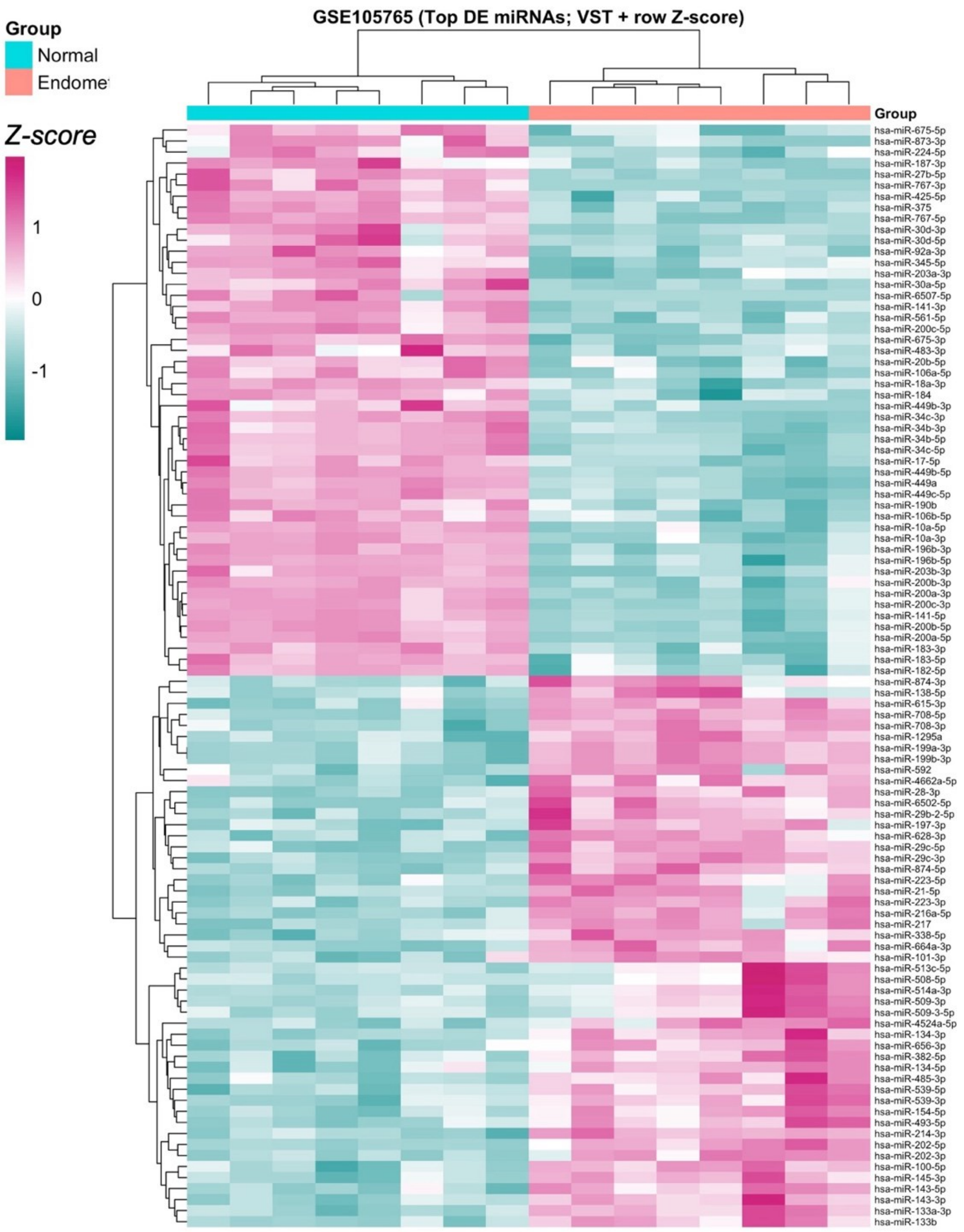

Figure S1. Heatmap of top differentially expressed miRNAs in the GSE105765 dataset. Heatmap showing the expression profiles of the top differentially expressed (DE) miRNAs between normal (n = 8) and endometriosis (n = 8) samples in the independent GSE105765 dataset. Differential expression analysis was performed using DESeq2, and the top 50 upregulated and top 50 downregulated miRNAs were selected based on adjusted p-value and $\log_2$ fold change thresholds. Count data were transformed using variance stabilizing transformation (VST) and scaled by row-wise Z-score normalization. Hierarchical clustering of both miRNAs and samples was performed using Euclidean distance with complete linkage. The color scale represents relative expression levels, where pink indicates higher expression and teal indicates lower expression.

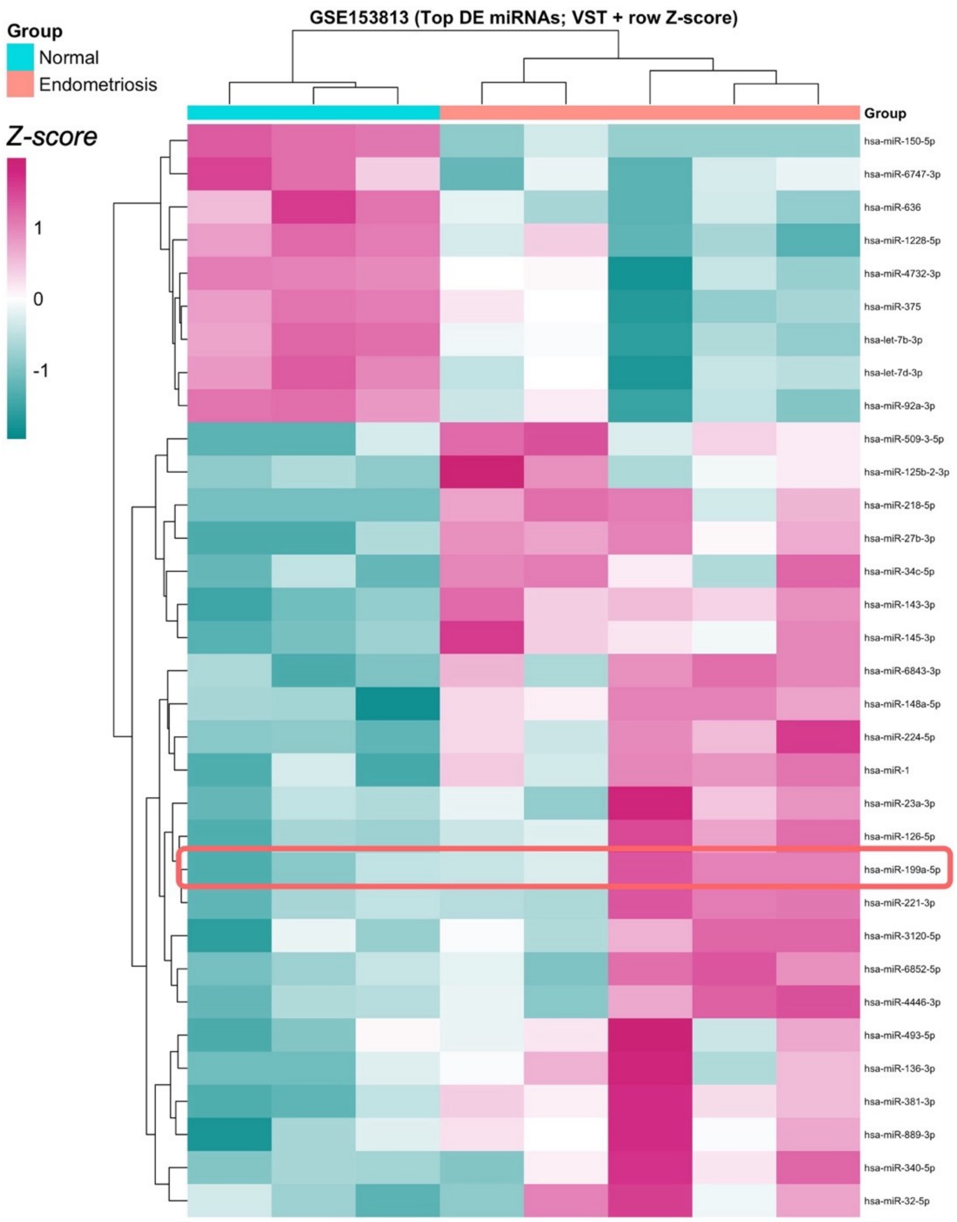

Figure S2. The heatmap of dysregulated miRNAs in GSE153813 dataset. Heatmap showing the expression profiles of significantly dysregulated miRNAs between normal and endometriosis samples in the GSE153813 dataset. Differential expression analysis was performed using DESeq2, and a total of 9 upregulated and 21 downregulated miRNAs were selected based on adjusted p-value and $\log_2$ fold change thresholds identified from the volcano plot. Count data were transformed using variance stabilizing transformation (VST) and scaled by row-wise Z-score normalization. Hierarchical clustering of both miRNAs and samples was performed using Euclidean distance with complete linkage. The color scale represents relative expression levels, where pink/red indicates higher expression and teal/blue indicates lower expression.

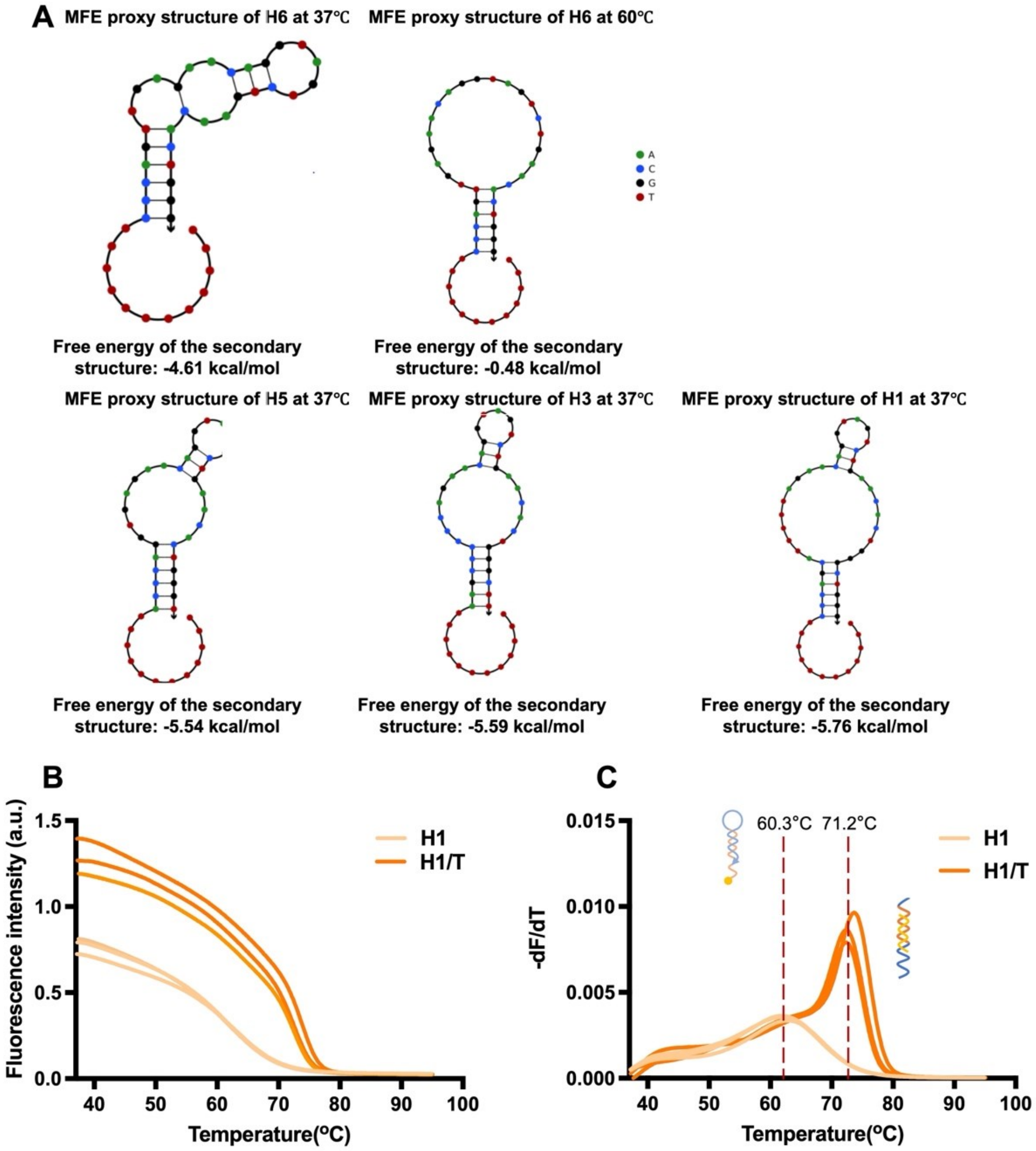

Figure S3. Rational design of probes. (A) NUPACK-predicted minimum free energy (MFE) structures of the designed hairpin probes, displaying their secondary structures and free energies (kcal/mol) for H1, H3, H5, and H6 at 37 °C, and for H6 also at 60 °C. A comparison of H6 at the two temperatures reveals a reduction in folding stability (ΔG from –4.61 kcal/mol to –0.48 kcal/mol) and the disappearance of its loop region, suggesting temperature-driven unfolding. This observation aligns with experimental results showing that H6 maintains higher DSN resistance at elevated temperatures due to conformational relaxation; (B) Melting curve and (C) melting peak analysis of H1 and H1/T duplex after SYBR Green staining. H1: hairpin probe. T: DNA-199a-5p.

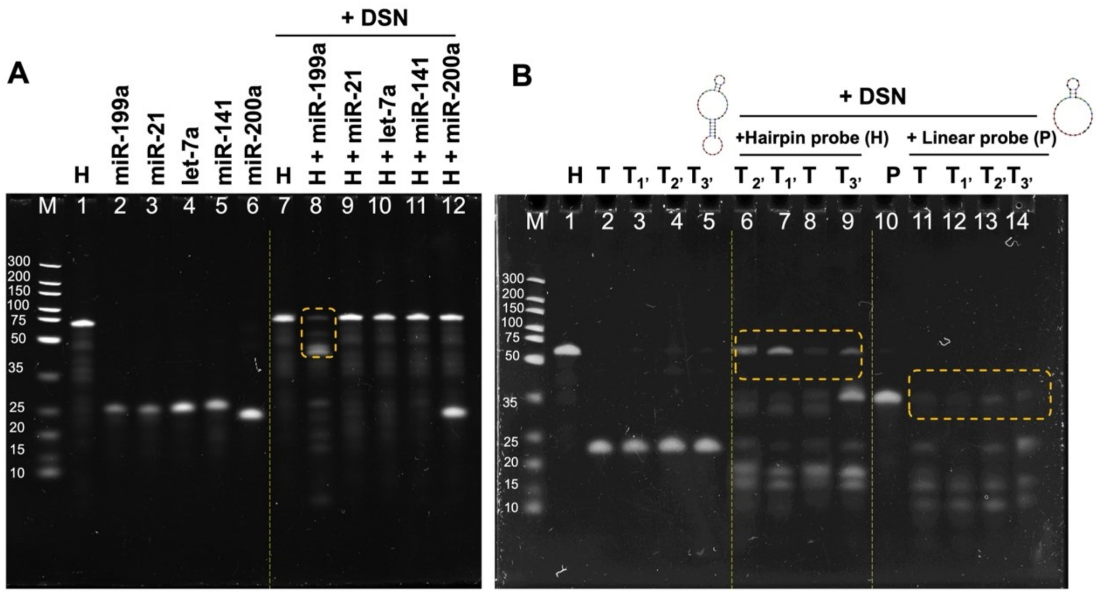

Name	Sequences (5'→3')
miR-21	UGA GGU AGU AGG UUG UAU AGU U
Let-7a	UAG CUU AUC AGA CUG AUG UUG A
miR-141	UAA CAC UGU CUG GUA AAG AUG G
miR-200a	UAA CAC UGU CUG GUA ACG AUG U

Name	Sequences (5'→3')
DNA-199a	CCC AGT GTT CAG ACT ACC TGT TC
DNA-199a 1-mutate ($T_{1'}$)	CCA AGT GTT CAG ACT ACC TGT TC
DNA-199a 2-mutate ($T_{2'}$)	CCA AGT GTT GAG ACT ACC TGT TC
DNA-199a 3-mutate ($T_{3'}$)	CCA AGT GTT GAG ACT ACA TGT TC

Figure S4. Characterization of the hairpin probe used in the DSN-assisted amplification reaction, analyzed by denaturing PAGE (15%). (A) DSN digestion of the hairpin probe in the presence of different miRNAs; (B) Comparison of DSN cleavage results using the hairpin and linear probes with targets containing one, two, or three mismatches. Denaturing PAGE was performed using a 15% polyacrylamide gel containing 7 M urea, with 0.5× TBE as the running buffer. Electrophoresis was conducted at 70 V for 85 min at 50 °C. M: marker; H: hairpin probe; T: target miR-199a.

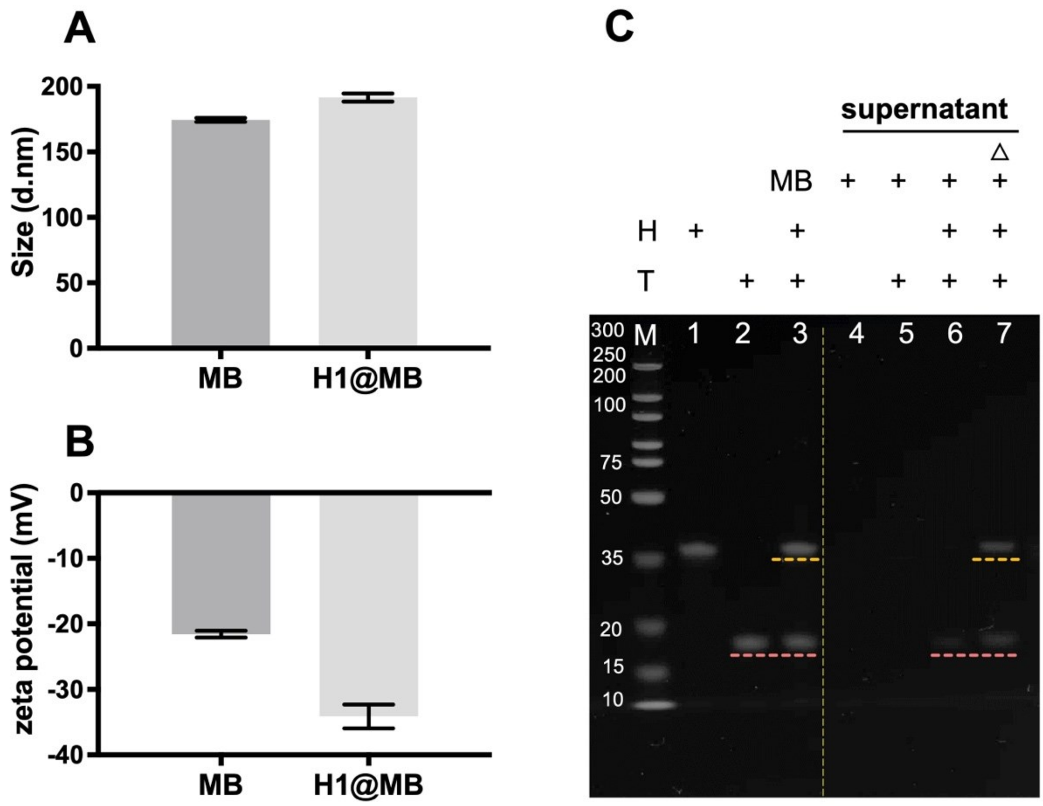

Figure S5. Characterization of MB-hDNA and bare MB by dynamic light scattering (DLS), Zeta potential analyzer, and gel electrophoresis. (A) Hydrodynamic diameter; (B) Zeta potential of of MB–hDNA and bare MB determined by Zetasizer; (C) Gel electrophoretic analysis of the hybridization reaction of MB-hDNA in the absence or presence of target miR-199a-5p. H: 200 nM hairpin-structured probe. T: 200 nM miR-199a-5p. Lane 4: supernatant of MB without hDNA addition. Lane 5: supernatant of MB after hDNA conjugation. Lane 6: supernatant of MB-hDNA after reaction with target miR- 199a. Lane 7: supernatant of the MB-hDNA/miR-199a-5p after heat treatment. M: DNA markers (10–300 bp). Electrophoresis was performed using a 15% polyacrylamide gel at 70 V for 120 min at 25 °C. Error bars represent the standard deviation of triplicate measurements (n=3).

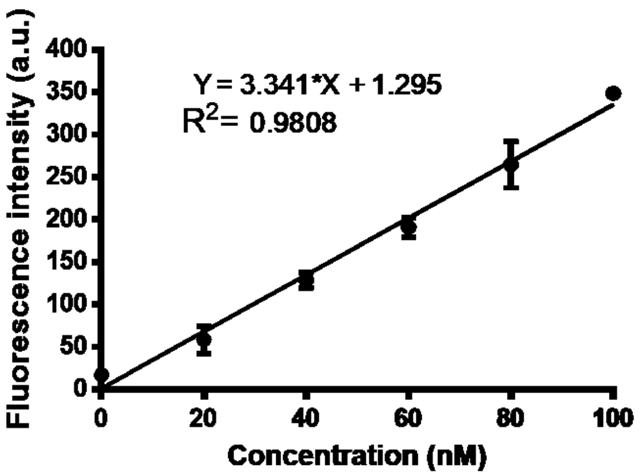

Figure S6. Calibration curve showing the relationship between hDNA concentration and corresponding fluorescence intensity following SYBR Gold staining. The fluorescence signal increased proportionally with increasing hDNA concentration, indicating a linear correlation between DNA quantity and SYBR Gold fluorescence. The excitation and emission wavelengths were set at λ_{ex}=490 nm and λ_{em}=520 nm, respectively. Error bars represent the standard deviation of triplicate measurements (n=3).

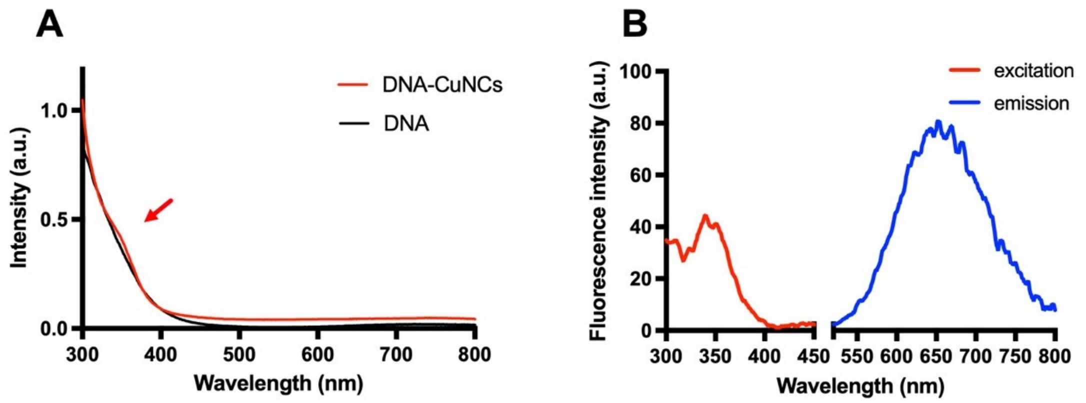

Figure S7. Characterization of copper nanoclusters (CuNCs) templated by poly(thymidine) (polyT) DNA. (A) UV-vis absorbance spectra; (B) excitation (λ_{em}=650 nm), and emission (λ_{ex} max=340 nm) spectra formed by 60-base polyT DNA (T60).

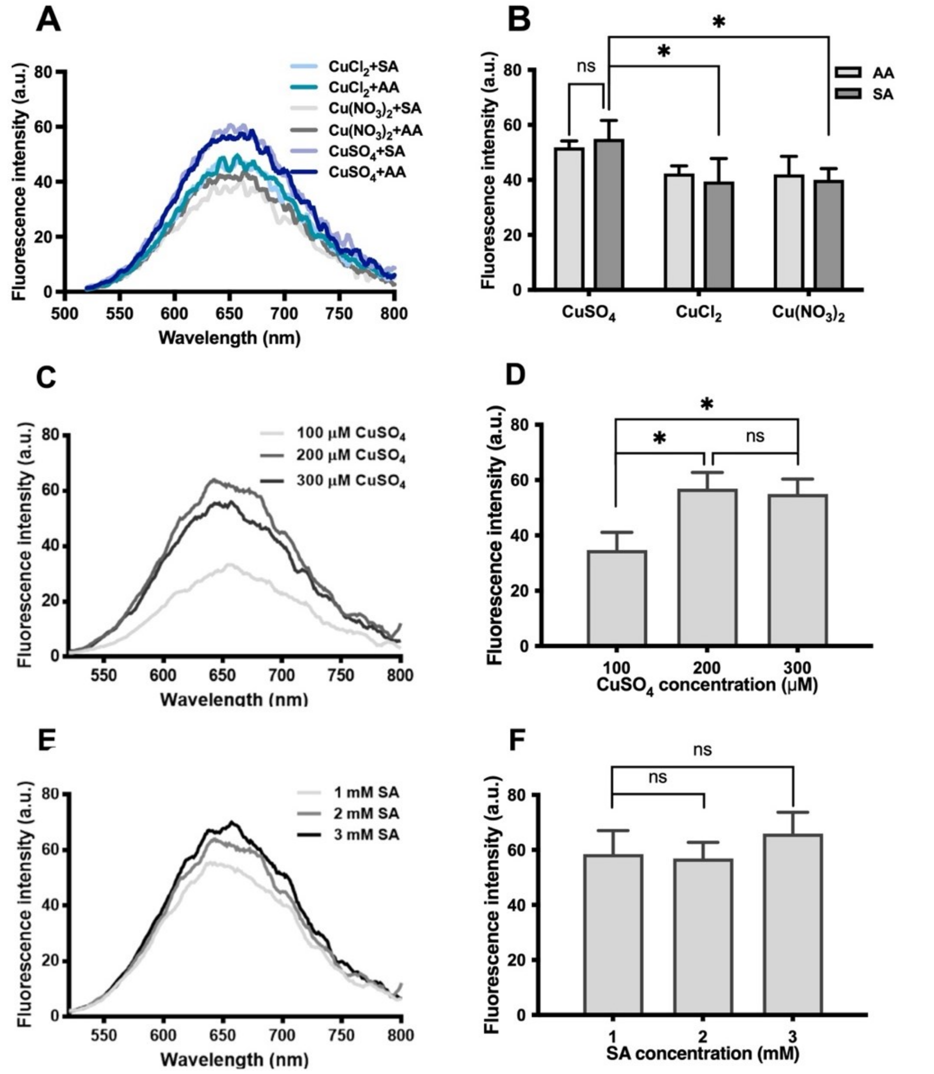

Figure S8. Optimization of the synthesis conditions for copper nanoclusters (CuNCs) templated by poly(thymidine) (polyT) DNA. (A) Fluorescence spectra of polyT-templated CuNCs prepared using copper sulfate, copper carbonate, or copper nitrate in combination with ascorbic acid or sodium ascorbate as reducing agents; (B) Maximum fluorescence intensities (λ_{em}=650 nm) of the samples in (A) at an excitation wavelength of 340 nm; (C) Fluorescence spectra of polyT-templated CuNCs synthesized with 2 mM sodium ascorbate and varying concentrations of copper sulfate (100, 200, and 300 μM); (D) Maximum fluorescence intensities (λ_{em}=650 nm) of the samples in (C) at an excitation wavelength of 340 nm; (E) Fluorescence spectra of polyT-templated CuNCs synthesized with 200 μM copper sulfate and varying concentrations of sodium ascorbate (1, 2, and 3 mM); (F) Maximum fluorescence intensities (λ_{em}=650 nm) of the samples in (E) at an excitation wavelength of 340 nm. SA: sodium ascorbate. AA: ascorbic acid. Statistical analysis was performed using student's *t*-test. ns: not significant, *: p <0.05. Error bars represent the standard deviation of triplicate measurements (n=3).

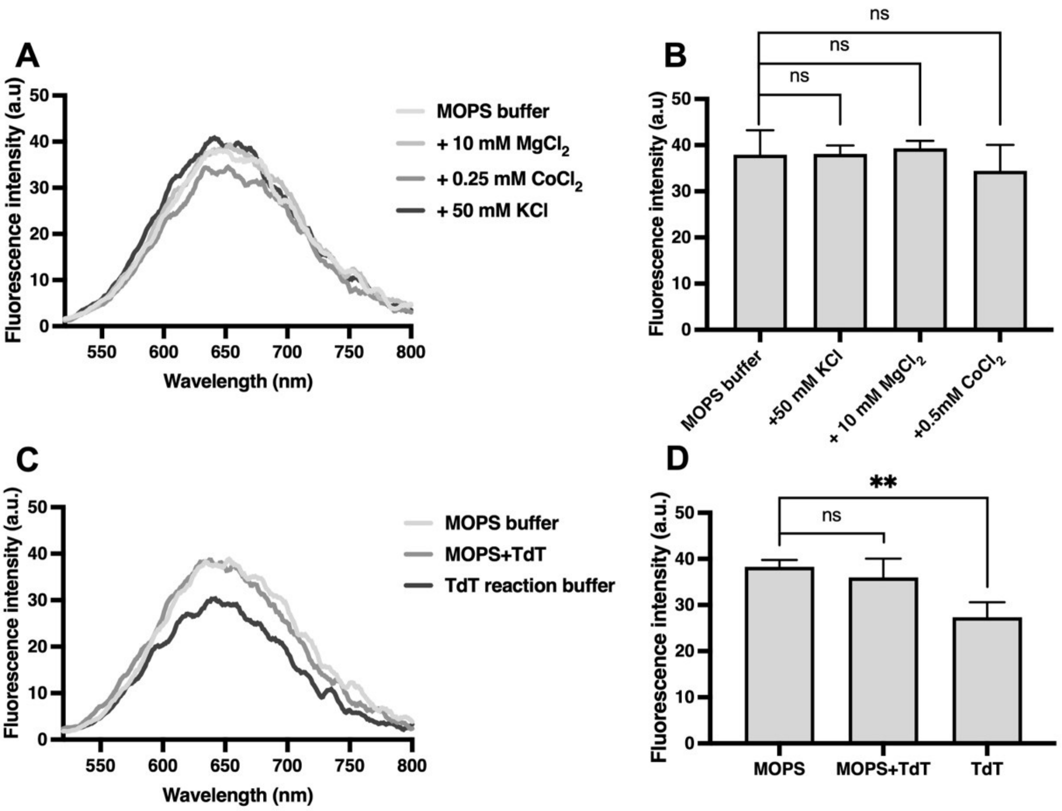

Figure S9. Effects of reaction-buffer cations and buffer composition on the fluorescence properties of poly(thymidine)-templated copper nanoclusters (polyT–CuNCs). (A) Fluorescence emission spectra of polyT–CuNCs synthesized in MOPS buffer and in MOPS buffer supplemented with 50 mM K^{+}, 10 mM Mg^{2+}, or 0.25 mM Co^{2+}; (B) Corresponding maximum fluorescence intensities of the samples shown in (A) under excitation at 340 nm; (C) Fluorescence emission spectra of polyT–CuNCs synthesized in MOPS buffer, in TdT reaction buffer, and in a mixture of MOPS buffer and TdT reaction buffer; (D) Corresponding maximum fluorescence intensities (λ_{em}=650 nm) of the samples shown in (C) under excitation at 340 nm. Statistical analysis was performed using student's *t*-test. ns: not significant; **: $p < 0.01$. Error bars represent the standard deviation of triplicate measurements.

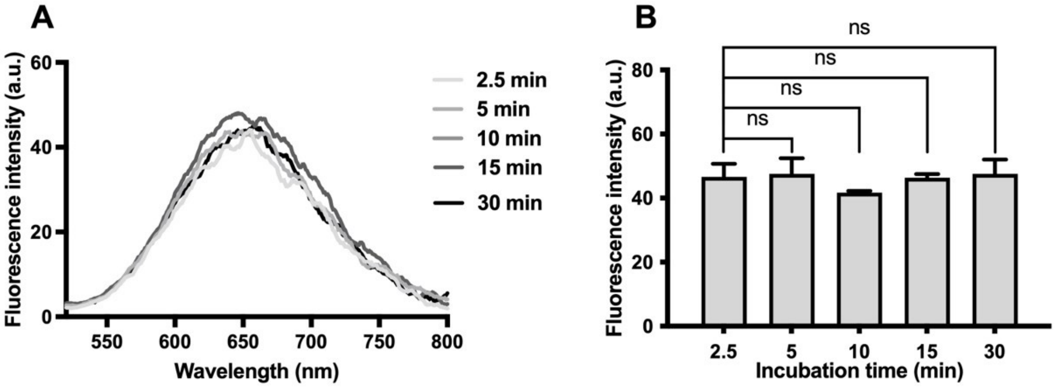

Figure S10. Fluorescence stability of poly(thymidine)-templated copper nanoclusters (polyT–CuNCs). (A) Fluorescence emission spectra of CuNCs formed by 60-base polyT DNA (T60) recorded after incubation for 2.5, 5, 10, 15, and 30 min under the optimized reaction buffer conditions; (B) Corresponding maximum fluorescence intensities (λ_{em}=650 nm) of the samples shown in (A), measured at the emission maximum under excitation at 340 nm. No statistically significant difference was observed among the fluorescence intensities at different incubation times (ns, not significant), indicating good fluorescence stability of polyT–CuNCs within the tested time window. Statistical analysis was performed using student's *t*-test. ns: not significant. Error bars represent the standard deviation of triplicate measurements.

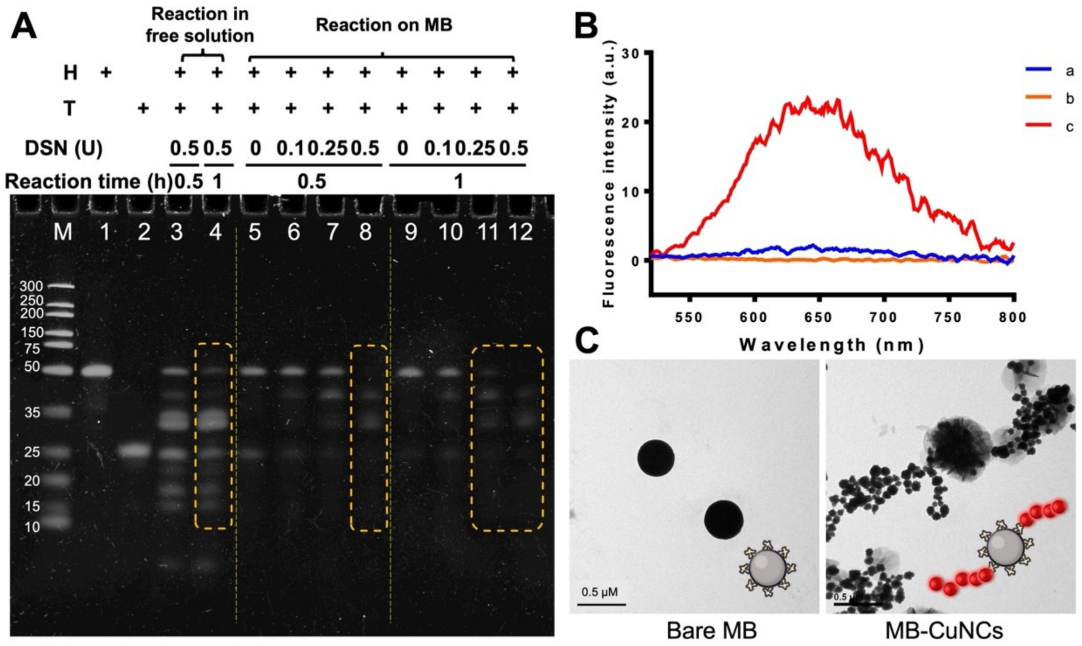

Figure S11. Feasibility Assessment of the Biosensing Platform. (A) Denaturing PAGE analysis comparing DSN digestion of MB–hDNA and free hDNA in the presence of target miR-199a. Denaturing PAGE: 15% acrylamide gel with 7 M urea. Electrophoresis conditions: 70 V, 85 min, 50 ºC. Running buffer: 0.5x TBE. M: Marker, H: hairpin probe; T: target miR-199a; (B) Fluorescence spectra of copper nanoclusters (CuNCs) produced by the TdT elongated, DSN-digested MB-hDNA; (C) Transmission electron microscopy (TEM) images showing the morphology of magnetic beads (MBs) under different conditions: the left panel displays bare MB, and the right panel shows MB decorated with CuNCs.

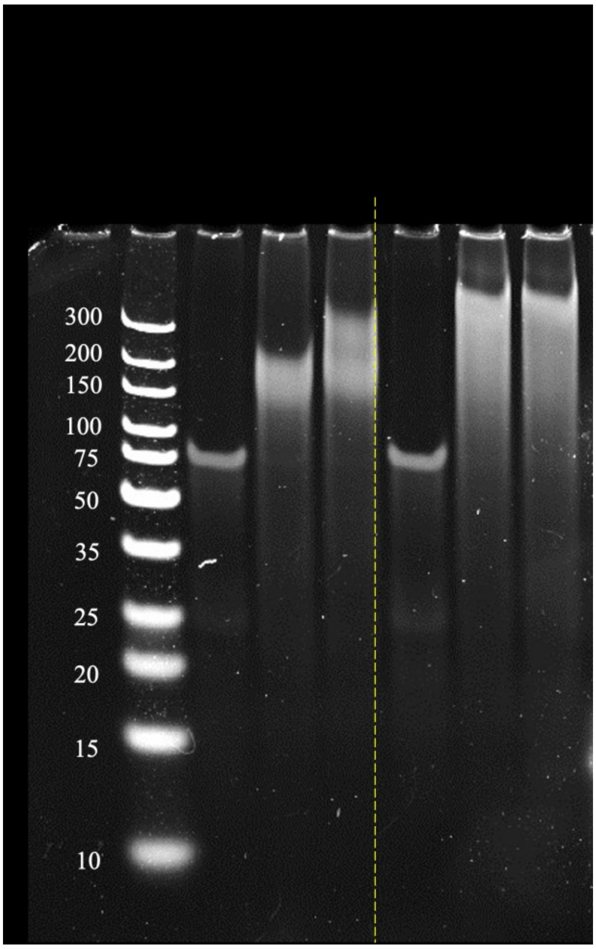

Figure S12. Characterization of TdT-mediated extension in free solution at different reaction times analyzed by PAGE. TdT reactions were carried out for 0.5, 1.5, 3 and 4.5 h in the presence of 200 nM template DNA, 4 mM dTTP and 10 U of TdT. PAGE analysis was performed using a 15% polyacrylamide gel with 0.5× TBE as the running buffer. Electrophoresis was conducted at 70 V for 120 min at room temperature. M: marker.

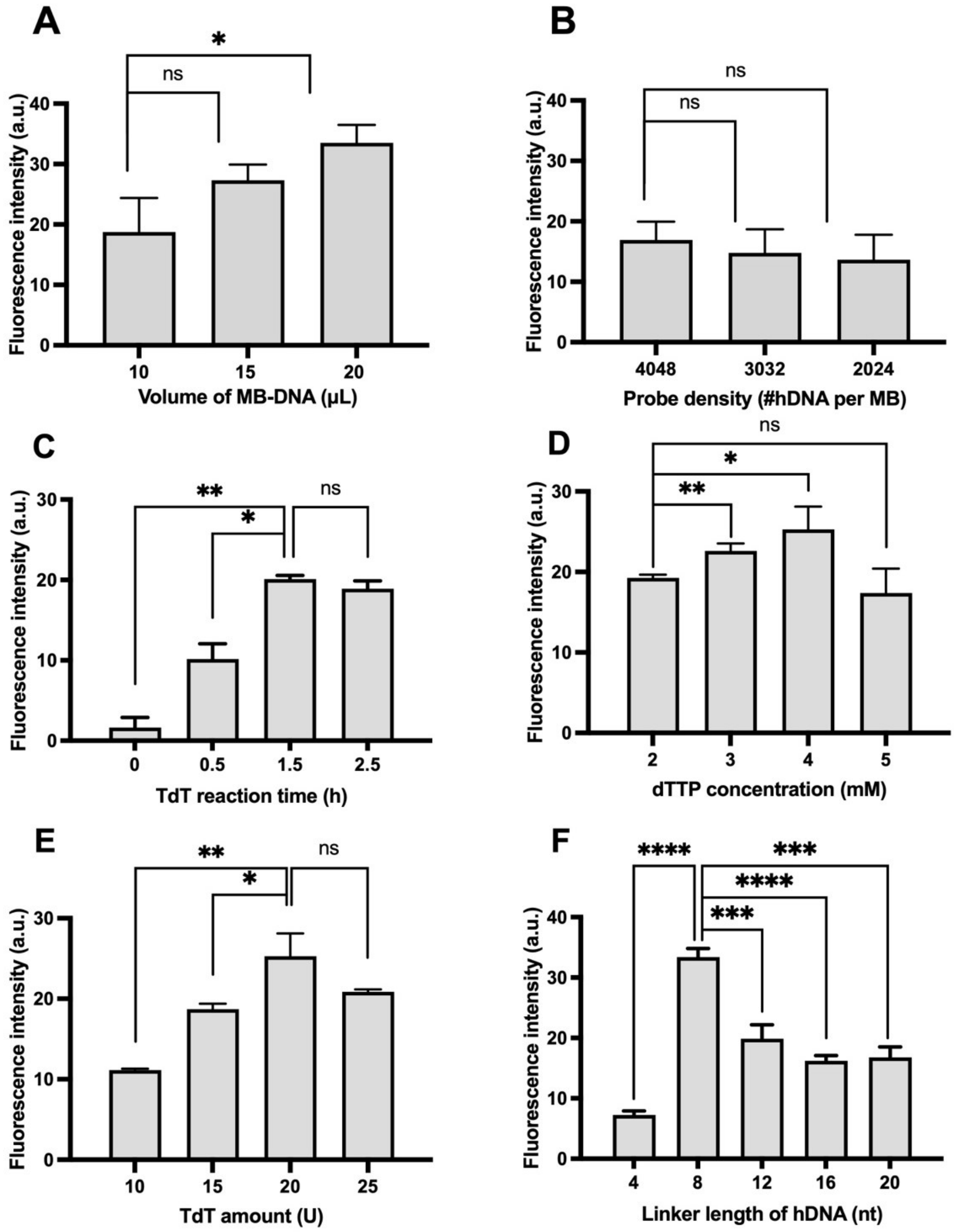

Figure S13. **Assay optimization.** (A) Effect of the volume of MB–DNA solution; (B) probe density; (C) reaction time; (D) dTTP concentration; (E) amount of TdT and linker length within hDNA using thymidine sequences of 4, 8, 12, 16, and 20 bases on the fluorescence intensity of CuNCs. Fluorescence spectra were recorded at an excitation wavelength of 340 nm (λ_{ex}=340 nm), scanned from 500 nm to 800 nm, with maximum emission observed at 650 nm (λ_{em}=650 nm). Statistical analysis was performed using Student's *t*-test. ns, not significant; *, $p < 0.05$; **, $p < 0.01$; ***, $p < 0.001$; ****, $p <$ 0.00001. Error bars represent the standard deviation of triplicate measurements (n=3).

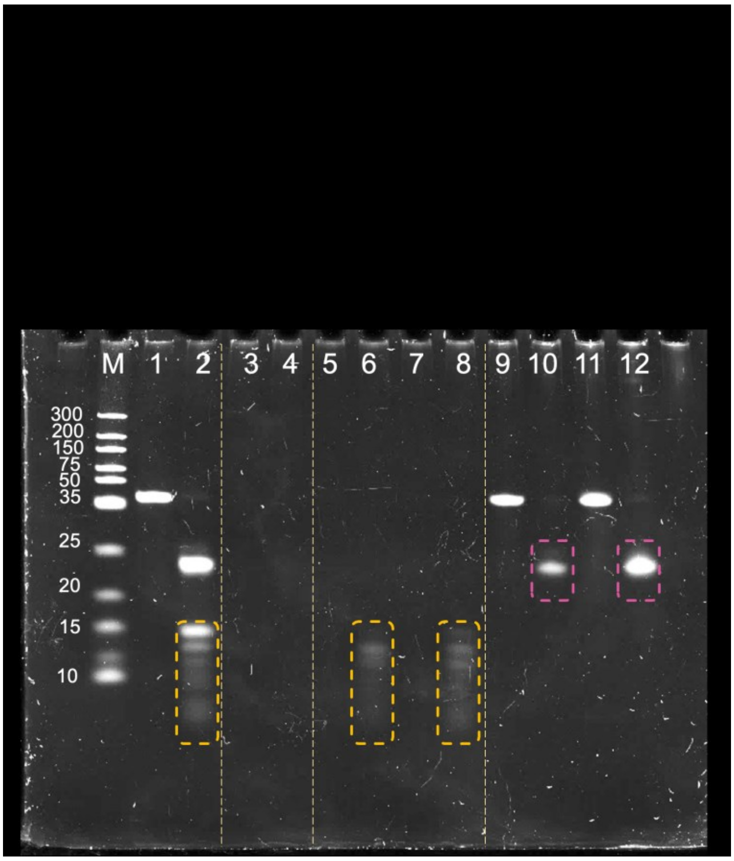

Figure S14. Characterization of hairpin probes immobilized at different probe densities (2024 and 4048 molecules of hDNA per bead) in the DSN-assisted amplification reaction on magnetic beads, analyzed by denaturing PAGE. Denaturing PAGE was performed using a 15% polyacrylamide gel containing 7 M urea, with 0.5× TBE as the running buffer. Electrophoresis was conducted at 70 V for 85 min at 50 °C. M: marker; H: hairpin probe; T: target miR-199a.

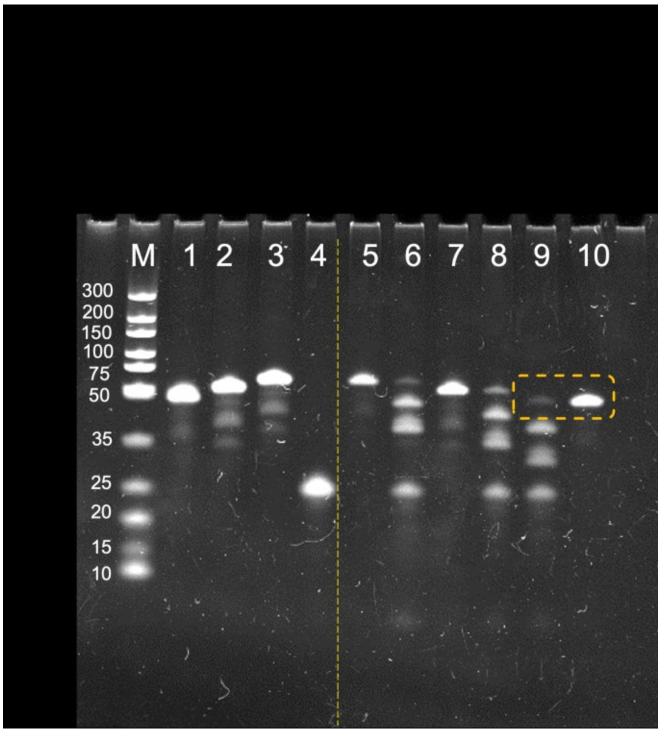

Figure S15. Characterization of hairpin probes containing 8-, 12-, and 16-thymidine linkers in the DSN-assisted amplification reaction on magnetic beads, analyzed by denaturing PAGE. Denaturing PAGE was performed using a 15% polyacrylamide gel containing 7 M urea, with 0.5× TBE as the running buffer. Electrophoresis was conducted at 70 V for 85 min at 50 °C. M: marker; H: hairpin probe; T: target miR-199a.

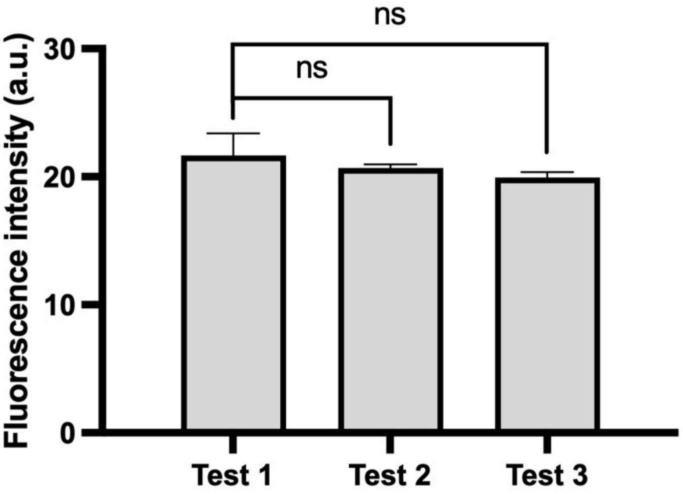

Intra-day			
	**Average**	**Std**	**CV (%)**
**Test 1**	21.67	1.71	7.90
**Test 2**	20.68	0.28	1.34
**Test 3**	19.94	0.43	2.16
**Inter-day**			
	**Average**	**Std**	**CV (%)**
**3 Tests**	20.77	0.87	4.18

Figure S16. Intra- and inter-day reproducibility of the DSN-TdT dual mode MB-hDNA assay. Fluorescence responses obtained from three independently prepared batches (Tests 1–3) at a representative target concentration. The excitation and emission wavelengths were set at λ_{ex}=340 nm and λ_{em}=650 nm, respectively. Bars represent the mean ± standard deviation (SD) for each batch (n=3). The accompanying table summarizes the intra-batch mean signal, standard deviation, and coefficient of variation (CV) for each batch, as well as the inter-batch CV calculated from the average signals of the three batches. Statistical analysis was performed using Student's *t*-test. ns: not significant.

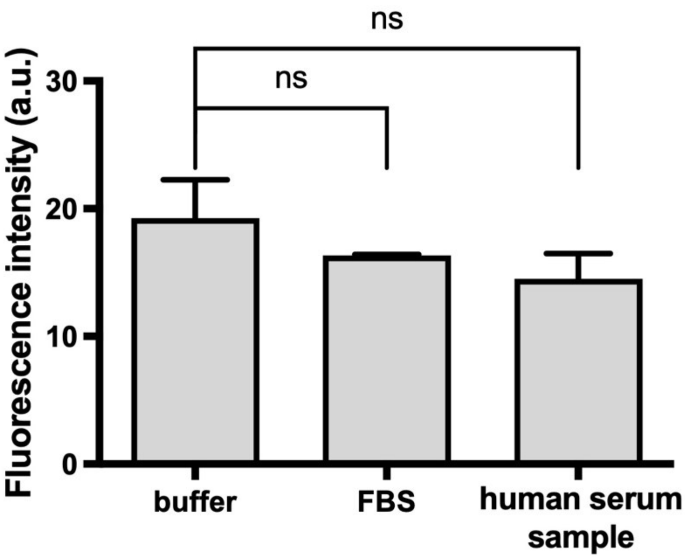

	Recovery Rate (%)	RSD (%)
**FBS**	92.49	0.49
**human serum sample**	84.54	8.7

Figure S17. Evaluation of matrix effects using a spiked-in sample in different biological media. Fluorescence intensity of two spiked-in samples (100 nM) in two different matrices, fetal bovine serum (FBS) and healthy human serum (λ_{ex}=340 nm; λ_{em} max=650 nm). The accompanying table summarizes the recovery rate and relative standard deviation (RSD) for each sample. Statistical analysis was performed using Student’s *t*-test. ns: not significant. Error bars represent the standard deviation of triplicate measurements (n=3).

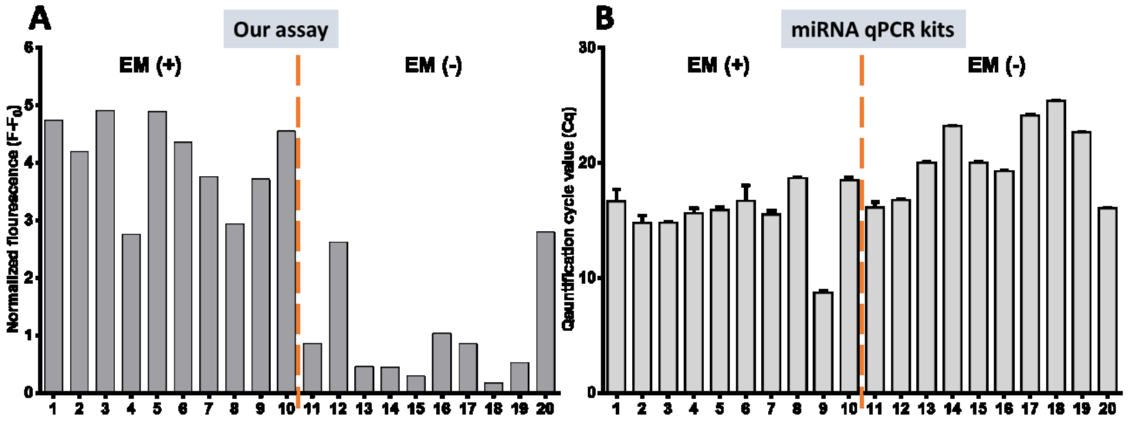

Figure S18. Quantitative analysis of the serum samples from individuals with endometriosis (EM+, N=10) and healthy controls (EM–, N=10). (A) Serum samples were analyzed using the proposed biosensing platform. λ_{ex}=340 nm; λ_{em} max=650 nm. Each sample was measured in duplicate; (B) Serum samples were analyzed using a commercial miRNA qRT-PCR kit. Error bars represent the standard deviation of triplicate measurements (n=3).

References

(1) Wang, Y.; Zhang, X.; Zhao, L.; Bao, T.; Wen, W.; Zhang, X.; Wang, S. Integrated amplified aptasensor with in-situ precise preparation of copper nanoclusters for ultrasensitive electrochemical detection of microRNA 21. *Biosens Bioelectron* **2017**, *98*, 386-391. DOI: 10.1016/j.bios.2017.07.009 From NLM Medline.

(2) Yuan, Y. H.; Chi, B. Z.; Wen, S. H.; Liang, R. P.; Li, Z. M.; Qiu, J. D. Ratiometric electrochemical assay for sensitive detecting microRNA based on dual-amplification mechanism of duplex-specific nuclease and hybridization chain reaction. *Biosens Bioelectron* **2018**, *102*, 211-216. DOI: 10.1016/j.bios.2017.11.030 From NLM Medline.

(3) Qi, Y. J.; Chen, Y.; Huang, Y.; Dou, B. T.; Zheng, S.; Li, Z. B.; Wang, P.; Zhang, S. K. Construction of an efficient microRNA sensing platform based on terminal deoxynucleotidyl transferase-mediated synthesis of copper nanoclusters. *Sensor Actuat B-Chem* **2025**, *424*. DOI: ARTN 136892

10.1016/j.snb.2024.136892.

(4) Zhou, Y.; Wang, H.; Zhang, H.; Chai, Y.; Yuan, R. Programmable Modulation of Copper Nanoclusters Electrochemiluminescence via DNA Nanocranes for Ultrasensitive Detection of microRNA. *Anal Chem* **2018**, *90* (5), 3543-3549. DOI: 10.1021/acs.analchem.7b05402 From NLM Medline.

(5) He, J. L.; Mei, T. T.; Tang, L.; Liao, S. Q.; Cao, Z. DSN/TdT recycling digestion based cyclic amplification strategy for microRNA assay. *Talanta* **2020**, *219*, 121173. DOI: 10.1016/j.talanta.2020.121173 From NLM Medline.

(6) Sadeghi, S.; Rahaie, M. Design and Fabrication of a DNA-copper Nanocluster-based Biosensor for Multiple Detections of Circulating miRNAs in Early Screening of Breast Cancer. *J Fluoresc* **2022**, *32* (6), 2297-2307. DOI: 10.1007/s10895-022-03023-z From NLM Medline.

(7) Li, Y.; Tang, D.; Zhu, L.; Cai, J.; Chu, C.; Wang, J.; Xia, M.; Cao, Z.; Zhu, H. Label-free detection of miRNA cancer markers based on terminal deoxynucleotidyl transferase-induced copper nanoclusters. *Anal Biochem* **2019**, *585*, 113346. DOI: 10.1016/j.ab.2019.113346 From NLM Medline.

(8) Borghei, Y. S.; Hosseini, M.; Ganjali, M. R. Fluorescence based turn-on strategy for determination of microRNA-155 using DNA-templated copper nanoclusters. *Microchim Acta* **2017**, *184* (8), 2671-2677. DOI: 10.1007/s00604-017-2272-6.
